# Copy number and transcriptome alterations associated with metastatic lesion response to treatment in colorectal cancer

**DOI:** 10.1002/ctm2.401

**Published:** 2021-05-01

**Authors:** Karen Gambaro, Maud Marques, Suzan McNamara, Mathilde Couetoux du Tertre, Zuanel Diaz, Cyrla Hoffert, Archana Srivastava, Steven Hébert, Benoit Samson, Bernard Lespérance, Yoo‐Joung Ko, Richard Dalfen, Eve St‐Hilaire, Lucas Sideris, Felix Couture, Ronald Burkes, Mohammed Harb, Errol Camlioglu, Adrian Gologan, Vincent Pelsser, André Constantin, Celia M.T. Greenwood, Sabine Tejpar, Petr Kavan, Claudia L. Kleinman, Gerald Batist

**Affiliations:** ^1^ Canadian National Centres of Excellence—Exactis Innovation 5450 Cote‐des‐Neiges Montreal Quebec H3T 1Y6 Canada; ^2^ McGill University‐Segal Cancer Centre, Jewish General Hospital 3755 Côte Ste‐Catherine Montreal Quebec H3T 1E2 Canada; ^3^ Charles LeMoyne Hospital 3120 Taschereau Blvd. Greenfield Park Quebec J4V 2H1 Canada; ^4^ Sacré‐Coeur de Montréal 5400 Boul. Gouin O Montreal Quebec H4J 1C5 Canada; ^5^ Sunnybrook Health Science Centre 2075 Bayview Ave. Toronto Ontario M4N 3M5 Canada; ^6^ St. Mary's Hospital 3830 Lacombe Montreal Quebec H3T 1M5 Canada; ^7^ Georges Dumont Hospital 220 Avenue Universite Moncton New Brunswick E1C 2Z3 Canada; ^8^ Hôpital Maisonneuve Rosemont 5415 Assumption Blvd Montreal Quebec H1T 2M4 Canada; ^9^ Hôtel‐Dieu de Quebec 11 Cote du Palais Montreal Quebec G1R 2J6 Canada; ^10^ Mount Sinai Hospital 600 University Avenue Toronto Ontario M5G 1X5 Canada; ^11^ Moncton Hospital 135 Macbeath Ave Moncton New Brunswick E1C 6Z8 Canada; ^12^ Gerald Bronfman Department of Oncology McGill University 3755 Côte Ste‐Catherine Montreal Quebec H3T 1E2 Canada; ^13^ Department of Epidemiology, Biostatistics and Occupational Health McGill University 3755 Côte Ste‐Catherine Montreal Quebec H3T 1E2 Canada; ^14^ Digestive Oncology Unit Katholieke Universiteit Leuven Oude Markt 13 Leuven 3000 Belgium; ^15^ Department of Human Genetics Lady Davis Research Institute, McGill University 3755 Côte Ste‐Catherine Montreal Quebec H3T 1E2 Canada

**Keywords:** colorectal cancer, copy number aberrations, metastasis, treatment response

## Abstract

**Background:**

Therapeutic resistance is the main cause of death in metastatic colorectal cancer. To investigate genomic plasticity, most specifically of metastatic lesions, associated with response to first‐line systemic therapy, we collected longitudinal liver metastatic samples and characterized the copy number aberration (CNA) landscape and its effect on the transcriptome.

**Methods:**

Liver metastatic biopsies were collected prior to treatment (pre, *n* = 97) and when clinical imaging demonstrated therapeutic resistance (post, *n* = 43). CNAs were inferred from whole exome sequencing and were correlated with both the status of the lesion and overall patient progression‐free survival (PFS). We used RNA sequencing data from the same sample set to validate aberrations as well as independent datasets to prioritize candidate genes.

**Results:**

We identified a significantly increased frequency gain of a unique CN, in liver metastatic lesions after first‐line treatment, on chr18p11.32 harboring 10 genes, including *TYMS*, which has not been reported in primary tumors (GISTIC method and test of equal proportions, FDR‐adjusted *p* = 0.0023). CNA lesion profiles exhibiting different treatment responses were compared and we detected focal genomic divergences in post‐treatment resistant lesions but not in responder lesions (two‐tailed Fisher's Exact test, unadjusted *p* ≤ 0.005). The importance of examining metastatic lesions is highlighted by the fact that 15 out of 18 independently validated CNA regions found to be associated with PFS in this study were only identified in the metastatic lesions and not in the primary tumors.

**Conclusion:**

This investigation of genomic‐phenotype associations in a large colorectal cancer liver metastases cohort identified novel molecular features associated with treatment response, supporting the clinical importance of collecting metastatic samples in a defined clinical setting.

## Graphical abstract

Graphical Abstract and Graphical Headlights

Liver metastatic lesions from colorectal cancer patients were collected before and after first‐line standard chemotherapy and comprehensively profiled with the objective to assess the genomic impact of systemic therapy and investigate association with response and survival. This study allowed identification of novel CNAs having an impact on the transcriptome with a potential prognostic value in patients with colorectal cancer.

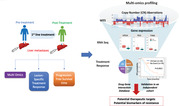

## 1 BACKGROUND

Colorectal cancer (CRC) patients with metastases have a relative 5‐year survival rate of 14% and the most common site of metastasis in CRC is the liver.^1^ Clinical responses of metastatic (m)CRC to first‐line treatment range from 15% to 60% but, unfortunately, patients almost inevitably develop therapeutic resistance.^2^ To date, molecular studies of CRC responsiveness to therapy have largely focused on primary tumors obtained at diagnosis, resulting in four consensus molecular subtypes predictive of outcome,^3^ a comprehensive copy number aberration (CNA) landscape with regions associated with drug response,^4^, ^5^ and a mutational profile that can be used to guide clinical practice for a few targeted therapies.^6^, ^7^ Recent studies characterizing CRC dynamics revealed that, most often, metastatic seeding occurs before diagnosis, when the primary carcinoma is clinically undetectable.^8^ While mutations in canonical driver genes (*KRAS*, *NRAS*, *BRAF*) are often concordant between primary and metastatic lesions,^9^, ^10^, ^11^ it has been reported that 15% and 19% of somatic mutations were primary tumor‐ and metastatic‐specific, respectively, suggesting a higher mutation rate in metastases and the possibility of genetic programs for site‐specific colonization.^12^ Since most novel therapies are tested in the metastatic setting, defining the molecular features of this target tissue is critical, since changes that occur over time, and during the course of prior therapy (eg, adjuvant) can affect the efficacy of subsequent lines of treatment. Serial tumor sampling through the disease trajectory can be instrumental to fully capture molecular evolution, identify biomarkers of disease progression, and develop strategies to select treatments in an optimal sequence to delay or overcome emerging resistance. Additionally, given the rapidly evolving work detecting specific genomic variants in cell‐free DNA (cfDNA) collected in liquid biopsies, the data in tissue that we and others generate is critical to expanding and validating the panel of cfDNA targets.^13^

Independent studies have shown consistent significant differences between primary tumors and metastatic lesions. A targeted sequencing strategy revealed that these differences were primarily at the CNA level.^14^ Discordance in mutational status has been documented in the clinically relevant *KRAS* gene,^15^, ^16^, ^17^, ^18^ and intrapatient intermetastatic lesion heterogeneity also appears to be a major feature of liver metastases, with a strong prognostic value.^19^ Furthermore, the consensus molecular subtypes defined in primary CRC have limited prognostic utility in the setting of oligometastatic disease where three distinct new molecular subtypes have been identified.^20^ Taken together, these results underscore the need to study metastatic lesions to assess the genomic impact of standard therapies, the genomic evolution of metastatic lesions over time of treatment and the implications for designing sequential treatments.

We and others have previously defined Next Generation Biobanking as a standardized collection of high‐quality serial biospecimens from patients in a specific clinical context.^21^, ^22^ To assess the molecular changes occurring during the course of treatment with a standard first‐line systemic therapy, we collected fresh‐frozen serial core biopsies, generating the largest banked set of longitudinal liver metastases profiled as comprehensively to date, and characterized them using Whole‐Exome Sequencing (WES) and RNA‐seq. The context of two sequential clinical trials provided the opportunity to collect these liver metastatic biopsy specimens before starting first‐line therapy (which we refer to as pre‐samples) and at the time of clinical progression (post‐samples), using small variations of universally accepted standard first‐line chemotherapy. Sixty‐eight percent of the patients received the oxaliplatin‐based regimen FOLFOX, or its chemically analogous combination with the oral formulation of 5‐FU in XELOX, while 22% received the irinotecan‐based regimen FOLFIRI, a clinically equivalent combination (essentially 5‐FU and leucovorin ), with the replacement of oxaliplatin with irinotecan. These combinations are the current universal choice chemotherapy combinations in the first‐line setting, and have essentially equivalent clinical efficacy in this setting. In most cases (70%), bevacizumab was also administered. We report here the molecular aberrations inferred at the CNA level, their transcriptomic impact, and their correlation with treatment response and with overall patient progression‐free survival (PFS).

## 2 METHODS

### 2.1 Patient cohorts

Samples were collected from mCRC patients enrolled in two concomitant clinical trials (NCT00984048/Q‐CROC‐01 and NCT01949194/Q‐CROC‐06) that were conducted from September 2009 until May 2018 at the same recruiting sites, using the same standard operational procedures (SOPs) and with the same first‐line treatments. Both studies were approved by the institutional review board at each participating hospital and complied with Good Clinical Practices, the principles of the Declaration of Helsinki and all applicable regulatory requirements. In both studies, enrolled patients had a confirmed mCRC diagnosis, were deemed unresectable at diagnosis, and had at least one liver metastatic site available for biopsy. All patients provided written informed consent for a biopsy before treatment and another biopsy (optional in NCT01949194) at disease progression as defined by Response Evaluation Criteria in Solid Tumors guideline version 1.0^23^ (RECIST 1.0). Patient information is summarized in Table 1. Paired pre‐ and post‐treatment liver metastatic lesions were collected for 21 patients. Among them, 16 received an oxaliplatin‐based regimen, 4 received irinotecan‐based regimen, and 1 received a combination of the two drugs.

**TABLE 1:** Baseline demographic and clinical characteristics

	QCROC‐01 (*N* = 136)		QCROC‐06 (*N* = 19)		Total (*N* = 155)	
Characteristics	*N*	%	*N*	%	*N*	%
**Age**						
Median	63	N/A	65	N/A	63	N/A
Min‐Max	39‐87	N/A	45‐88	N/A	39‐88	N/A
**Sex**						
Female	56	41%	3	16%	59	38%
Male	80	59%	16	84%	96	62%
**Ethnicity**						
Caucasian	124	91%	21	100%	145	92%
Black or African American	4	3%	0	0%	4	3%
Asian	3	2%	0	0%	3	2%
Aboriginal	1	1%	0	0%	1	<1%
Hispanic	1	1%	0	0%	1	<1%
Mauritius	1	1%	0	0%	1	<1%
Unknown	2	1%	0	0%	2	1%
**ECOG**						
0	52	38%	2	11%	54	35%
1	59	43%	16	84%	75	48%
2	6	4%	0	0%	6	4%
Unknown	19	14%	1	5%	20	13%
**Stage**						
IVa	59	43%	5	26%	64	41%
IVb	77	57%	14	74%	91	59%
**Primary site**						
Adenocarcinoma	127	93%	18	95%	145	94%
Mucinous adenocarcinoma	3	2%	1	5%	4	3%
Tubulovillous adenoma	2	1%	0	0%	2	1%
Neuroendorine/adenocarcinoma	1	1%	0	0%	1	<1%
Unknown	3	2%	0	0%	3	2%
**Tumor sidedness**						
Left	87	64%	13	68%	100	65%
Right	39	29%	5	26%	44	28%
Unknown	10	7%	1	5%	11	7%
**# Metastatic sites**						
1	59	43%	4	21%	63	41%
2	42	31%	6	32%	48	31%
>3	35	26%	9	47%	42	28%
**Best response**						
CR	1	1%	N/A	N/A	N/A	N/A
CR/resection	9	7%	N/A	N/A	N/A	N/A
PR	54	40%	N/A	N/A	N/A	N/A
SD	41	30%	N/A	N/A	N/A	N/A
PD	12	9%	N/A	N/A	N/A	N/A
Unknown	19	14%	N/A	N/A	N/A	N/A
**Bevacizumab**						
+	99	73%	10	53%	109	70%
–	29	21%	9	47%	38	25%
N/A*	8	6%	0	0%	8	5%
First‐line regimen						
Oxaliplatin‐based	97	71%	9	47%	106	68%
Irinotecan‐based	26	19%	8	42%	34	22%
Other	5	4%	2	11%	7	5%
N/A	8	6%	0	0%	8	5%
Metastatic KRAS mutation status						
+	56	41%	8	42%	64	41%
–	54	40%	11	58%	65	42%
N/A	26	19%	0	0%	26	17%

* Patient died before treatment start or only had a post‐biopsy.

N/A, not applicable.

### 2.2 Objective response of liver metastasis lesions

The decision to declare patient's clinical resistance to treatment was based on classical RESIST criteria, which uses cumulative measurements of all lesions. A clinical resistance triggered rebiopsy of the lesion, and a change in treatment. As it happens, so‐called “mixed responses” appeared to be more common than expected, so that there were patients with overall clinical progression, in which the initially biopsied, and/or rebiopsied lesion was actually responding to treatment. Hence the opportunity to examine some lesion‐specific partial responders.

When multiple metastatic lesions were observed pre‐treatment, the targeted biopsy was chosen based on feasibility of a safe and successful biopsy by the interventional radiologist. The same lesion was rebiopsied after treatment, when feasible. RECIST 1.0 was used as a guideline for evaluating lesion specific responses (defined as the size of the post‐treatment lesion compared to its size at baseline) and were categorized as either intrinsic resistant (IRES), lesions that continued to grow despite the therapy, partial responder (PR), stable (SD) or acquired resistant (ARES), where an initial documented response was eventually followed by regrowth of the tumor. More specifically, a lesion was considered IRES if its longest diameter increased by ≥20% by the first 8‐week evaluation; a lesion was categorized as PR when a ≥30% decrease in the largest diameter since start of treatment was observed using the baseline measurement as reference; a lesion with a < 30% decrease in the longest diameter taking as reference the baseline measurement or a <20% increase in the longest diameter taking as reference the smallest diameter was considered as SD; and a lesion with a PR or SD response followed by a ≥20% increase in the longest diameter taking as reference the smallest diameter was characterized as ARES.

### 2.3 Tumor sample collection, processing, and quality assessment

Three percutaneous ultrasound guided needle core liver metastasis biopsies were performed for each patient as described.^22^ The first two were either snap frozen in liquid nitrogen or immersed in RNALater solution while the third sample was placed in 10% neutral buffered formalin for hematoxylin and eosin staining (H&E) (see Supplemental Methods). Blood samples were collected at baseline for each patient and genomic DNA was isolated from buffy coat. Genomic material isolation was performed as previously reported using the AllPrep Universal Kit (Qiagen) for FF and RNAlater samples^22^ or using the QIAamp FFPE extraction kit (Qiagen) for FFPE samples. Germline DNA was extracted from buffy coat or whole blood using the Gentra PureGene Blood kit (Qiagen) according to manufacturer's instructions (see Supplemental Methods for quality‐control metrics).

### 2.4 Whole exome sequencing and copy number aberration data analysis

Tumor and normal DNA samples were profiled by WES in five batches using SureSelect Exome V5 or V6 + UTR library capture (Agilent, USA) and sequenced on Illumina HiSeq 2500 or HiSeq 4000 (see Supplemental Methods for quality‐control metrics). Duplicate reads and PCR artifacts were removed using Picard Tool function MarkDuplicates before import into Nexus Copy Number™ (version 8.0, BioDiscovery, CA, USA) for CNA analysis. For each tumor sample, the matched normal DNA from blood of the same patient was used in the ngCGH (matched) processing according to the software instructions. For two liver metastatic and four primary tumor samples, normal matched DNA was not available, and they were processed using a pooled reference that was built with the normal DNA samples of the remaining patients using the BAM Reference Builder utility and according to the recommendations of the manufacturer (BioDiscovery Inc, CA, USA). SNP‐FASST2 Segmentation Algorithm was applied using defaults settings (see Supplemental Methods for settings and quality‐control metrics). Sex chromosomes were excluded from all analyses.

### 2.5 Statistical analysis of CNA

Areas of the genome with a statistically high frequency of CNA compared to normal controls (*Q*‐bound value ≤ 0.05 and *G*‐score cut‐off ≤ 1) corrected for multiple testing using FDR correction (Benjamini & Hochberg) were identified using the GISTIC^24^ tool approach in Nexus Copy Number software (BioDiscovery, CA, USA). The comparison of GISTIC CNA frequencies in pre‐ versus post‐sample groups was performed using either the test of equal proportions for unmatched samples or McNemar's test for the matched pair samples, and results were considered significant if multiple testing adjusted *p* value was ≤ 0.05. CNA frequency comparison analyses were also performed using a two‐tailed Fisher's Exact test in Nexus Copy Number software for unmatched samples. CNA region frequencies were considered significantly different at unadjusted *p*‐value thresholds of 0.005.

The log‐rank statistic^25^ was used to identify regions yielding a high degree of PFS association (permutated *p* ≤ 0.005). The *p* value is calculated by permuting the PFS time for each sample and comparing the log‐rank statistic for the permuted data to the original data. To compare survival times between two groups, Kaplan‐Meier curves were generated, and *p* values were computed using the log‐rank test. Visualization of CNA based on *p* value was generated using the *gaiaCNVplot* function from the “*TCGAbiolink*s” R package.

### 2.6 RNA sequencing and integration with copy number aberrations data

RNA samples (RIN > 3) were sequenced in four batches. RNA sequencing was performed using Illumina TruSeq Stranded mRNA or Illumina TruSeq stranded total RNA with RiboZero library preparation and sequenced on Illumina HiSeq 2500 or HiSeq 4000 (See Supplemental Methods for quality‐control metrics). Significant CNA regions from group comparisons and PFS analysis were selected to perform integration with expression data. Fold change (FC), *p* value (negative binomial test), and false discovery rate (FDR) were computed between the samples with the event and the ones without the event. Concordance between copy number and gene expression was considered true if the average and median gene expression minimal value were >1 RPKM, FC >1.5, and the FDR <0.1.

## 3 RESULTS

### 3.1 Cohort characteristics, clinical data, and sample analysis

**FIGURE 1:**
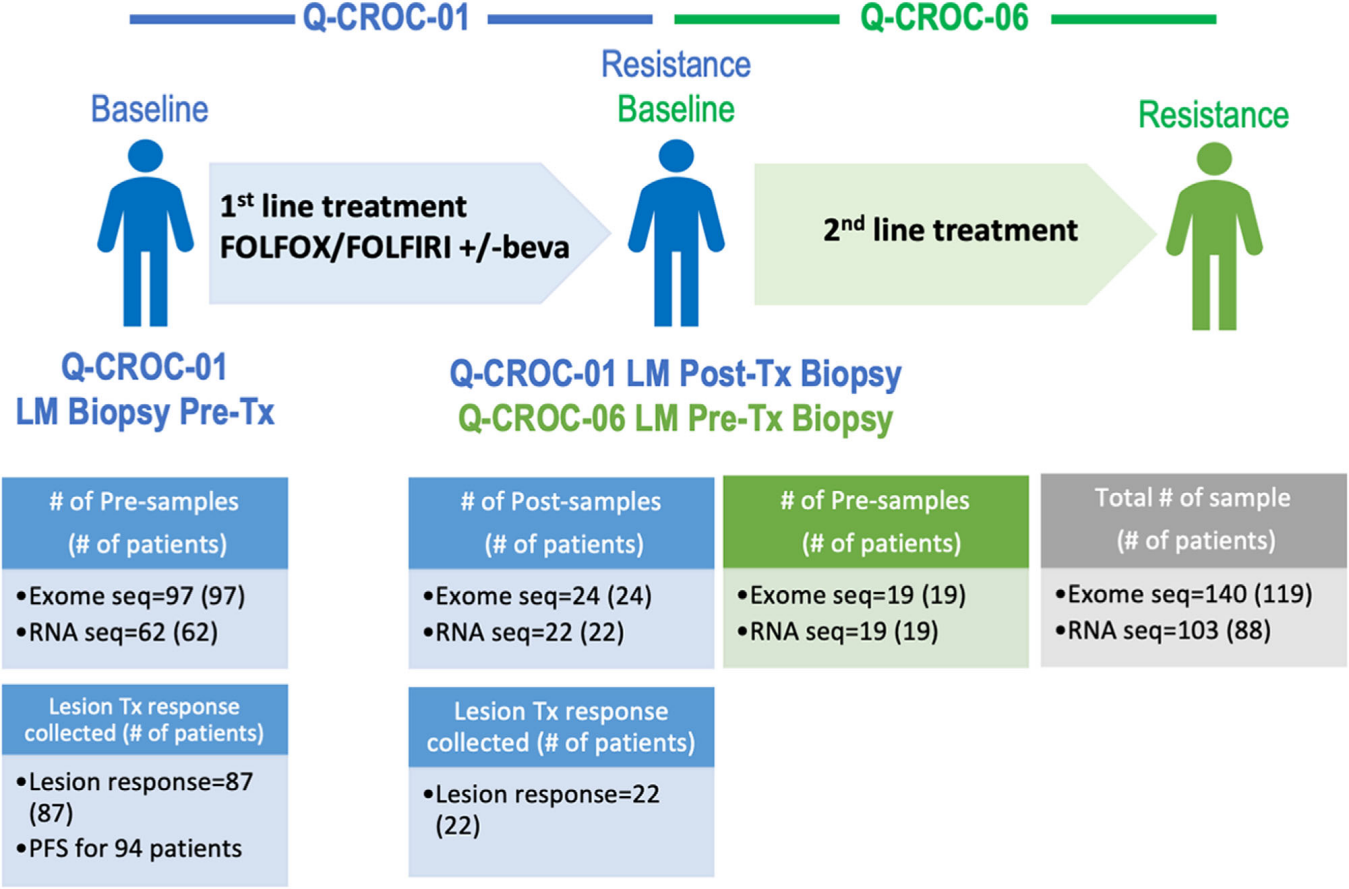
Q‐CROC‐01 and Q‐CROC‐06 sequential clinical trials and profiled samples. Q‐CROC‐01 and Q‐CROC‐06 clinical trials enrolled mCRC patients at the same sites. In Q‐CROC‐01, pre‐ (baseline) and post‐treatment (at clinical resistance) samples of liver metastasis lesions were profiled using WES and RNA sequencing. In Q‐CROC‐06, pre‐second‐line treatment samples of liver metastatic lesions were analyzed using the same platforms and were grouped with Q‐CROC‐01 post‐samples in subsequent pre‐ versus post‐group comparison analysis. When possible, lesion specific responses were measured using RECIST v.1.0 and PFS were collected for patients enrolled in Q‐CROC‐01 trial. LM: liver metastasis; Tx: treatment; #: number; Pre: pre‐treatment, Post: post‐treatment; beva: bevacizumab

Samples were collected from 136 mCRC patients enrolled on the observational Q‐CROC‐01 trial (Figure S1), with a median PFS of 9.7 months. Known clinical prognostic parameters were tested for PFS associations and only disease stage IVb was identified as a poor prognostic marker compared to stage IVa (*p* = 0.03; Table S1). A sequential second‐line trial, Q‐CROC‐06, enrolling mCRC patients, with who had progressed on the very same standard first‐line therapies, that is, essentially identical clinical cohort (Figure 1), was leveraged to increase the post first‐line sample cohort size by 19 additional liver metastatic post‐biopsy samples. After passing all quality control criteria, our standardized collection of longitudinal liver metastatic samples comprised 140 samples profiled by WES (97 at baseline, pre‐samples, and 43 at progression, post‐samples), and 103 profiled by RNA sequencing (62 pre‐samples, 41 post‐samples) (Figures 1 and S1).

### 3.2 Copy number variation landscape of liver metastasis samples

We inferred the genome‐wide CNA landscape of all metastatic samples profiled by WES. We first validated our CNA inference workflow by applying it to previously reported SNPs^19^, ^26^ and CGH array^5^ datasets, and verified that our workflow reproduced the frequencies reported (Figure S2A). Next, we compared the performance of WES to the widely used CytoScan HD platforms for CNA inference, which we generated from a subset of 45 liver metastatic DNA samples, obtaining a similar CNA landscape and an equivalent percentage of genome change (Figures S2B‐H). Overall, CNA analysis from the WES data were concordant with the gold standard SNP‐array technology and provided better coverage for the coding region of the genome. Thus, we proceeded to analyze the rest of the cohort using WES data.

**FIGURE 2:**
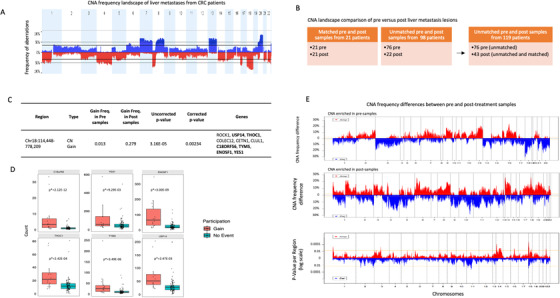
CNA profiles of CRC liver metastatic lesions and influence of chemotherapy treatment. (A) Aberration frequency plot of 119 liver metastatic lesions from 119 patients. *y* Axis shows frequency of gains (above 0 in blue) and losses (below 0 in red) and are shown as a function of chromosome region (*x* axis). (B) Among the 119 patients, 21 had paired pre‐ and post‐liver metastatic samples profiled. The CNA landscape of pre‐ and post‐liver metastatic lesions was compared using 119 samples (1 sample per patient and unmatched 76 pre‐ and 43 post‐samples). (C) CNA region showing significantly different frequencies between unmatched pre‐ and post‐treatment metastatic samples. In bold: genes showing a correspondence between CNA and gene expression. (D) Correspondence between copy number change and gene expression in 6 genes on chr18p11.32 is shown on box plots inferred from RNAseq data (count value). p*: FDR‐adjusted *p* value. (E) CNA frequency differences (*y*‐axis) between pre‐ and post‐samples are plotted as a function of chromosome region (*x*‐axis). Top and middle panels show CNA (gains in red, losses in blue) enriched in pre‐ and post‐samples, respectively. Bottom panel shows *p* values of frequency difference of gains and losses between the two groups as a function of chromosome region (*x*‐axis). Pre: pre‐treatment, Post: post‐treatment

Overall, at the chromosomal arm level, the CNA landscape matched those reported by the TCGA^6^ and the CAIRO^5^ studies for primary CRC samples and also by Sveen et al^19^ for liver metastatic samples (Figure 2A). We identified 30 significant (Q‐bound < 0.05) focal aberrations using the GISTIC method^24^ (Figures S3A and Table S2). Eight and 10 out of these 30 CNA regions overlap with GISTIC‐significant aberrations found in CAIRO2 and TCGA cohorts, respectively. Seventeen focal aberrations, including a CN gain covering the protooncogene *MET* on chr 7q31.2, are unique to our cohort (Figure S4).

### 3.3 Liver metastases CNA landscape and influence of standard chemotherapy

CNA divergence between pre‐ and post‐treatment metastatic samples was evaluated to assess the impact of chemotherapy exposure on these lesions, regardless of documented response of the lesions to treatment. Matched and unmatched pairs were analyzed separately (Figure 2B). The GISTIC method was applied to the samples in both pre‐ and post‐groups to identify recurrent CNA regions within each group, and the difference in frequencies between the two groups was assessed. Matched pair analyses showed no significant differences between pre‐ and post‐samples (*n* = 21, McNemar's test). On the other hand, the analysis of unmatched pairs revealed a unique region of gain on chr18p11.32 containing 10 genes, significantly enriched in the post‐treatment group compared to pre‐treatment samples (27.9% in post‐ vs 1.3% in pre‐samples; FDR corrected *p* < 0.05, test of equal proportions; Figure 2C). Six of these showed significant association between transcriptional expression level and CNA (*USP14*, *THOC1*, *C18orf56*, *TYMS*, *ENOSF1*, and *YES1)*, indicating a possible direct effect of CNA on the transcriptome (negative binomial test, fold change > 1.5, FDR‐adjusted *p* < 0.1, Figure 2D), which provides some validation of the significance of these findings. These results are consistent with previous findings showing that copy number variation mechanisms led to the regulation of *TYMS* expression level,^27^ a well‐characterized gene involved in 5‐FU resistance.^28^ The GISTIC method, which is very conservative, limited the number of focal regions tested in this analysis to 63 through the entire genome. We thus expanded our investigation with an alternative approach, identifying all focal CNA regions in the unmatched cohort using the SNP‐FASST2 Segmentation Algorithm in Nexus Copy Number Software (*n* = 29,736). We used the group comparison tool based on a two‐tailed Fisher's Exact test to identify differences in CNA frequencies between unmatched pre‐ and post‐samples (Figure 2E). To control our family‐wise error rate at 5%, the Bonferroni threshold for the number of CNAs tested would have required a significance level of 1.7E‐6, and we did not achieve this significance level at any CNA. The sample size of this study, though larger than most that have been generated in a defined therapeutic setting, was still too small to achieve statistical significance. For example, for a 30% difference in the rate of observing a CNA (10% vs 40%), to have 80% power we would need over 260 patient samples (see Supplementary Methods for Bonferroni threshold and sample size calculation for subsequent group comparison). We thus chose to report results for CNA region frequencies that we identified as different at unadjusted *p* thresholds of 0.005 on a two‐tailed Fisher's exact test. We do recognize that all these results must be interpreted cautiously and will require future independent validation, but the challenge of obtaining uniformly managed biopsies over time makes it important to report these findings to stimulate further work. Using this statistical approach, we found that 28 CN gains and 16 CN loss regions, containing a total of 437 genes, showed significant differences in frequencies between unmatched pre‐ and post‐treatment samples (unadjusted *p* ≤ 0.005, Figure 2E and Table S3). The gain region on chr18p11.32 identified using the GISTIC method was also found more frequently in post‐ versus pre‐samples in this comparison (22.3% in post vs 1.2% in pre, unadjusted *p* = 0.00058). At the gene level, we found a positive concordance between CNA and gene expression for 49 genes, including genes known or predicted to interact with drugs based on the Drug‐Gene Interaction database (DGIdb^29^) such as *SMAD2*, *PGF*, *TGFB3*, *ESR2*, *LAMA1*, *USP14*, *TYMS*, and *YES1*. This provides validation that the CNAs are expressed and therefore clinically relevant and also reinforces our analytic approach to this data. The list of 231 classic cosmic genes (https://cancer.sanger.ac.uk/cosmic) was also interrogated for significant differences (Fisher's test, FDR corrected *p* < 0.05) in CNA frequencies between the two groups and 16 genes were identified (*BRCA2*, *CDKN2A*, *RB1*, *FOXA1*, *GNAS, HIF1A, MAX, NT5C2, RAD21, SIX1, GATA3, SUFU, TSHR, MMYOD1, PAX5*, and *WT1*) (Table S4). A CN gain covering *TSHR* exhibited a significantly higher frequency in post‐samples compared to pre‐samples, with a significant overexpression in patients harboring the aberration, again providing evidence that the CNA results in altered expression, at least at the transcriptional level. Overall, this genome‐wide comparison of the CNAs in liver metastases, before and after a clinical exposure to chemotherapy, allowed us to identify genomic variations having a direct impact on the transcriptome. Somatic mutation profiles of pre‐ and post‐treatment metastatic samples were also compared but revealed no significant change in variant frequency between the two groups, which confirms the results already reported in the literature suggesting that FOLFOX regimen does not induce or select for new driver mutations^30^ (Table S5).

### 3.4 Copy number variation association with lesion objective response

**FIGURE 3:**
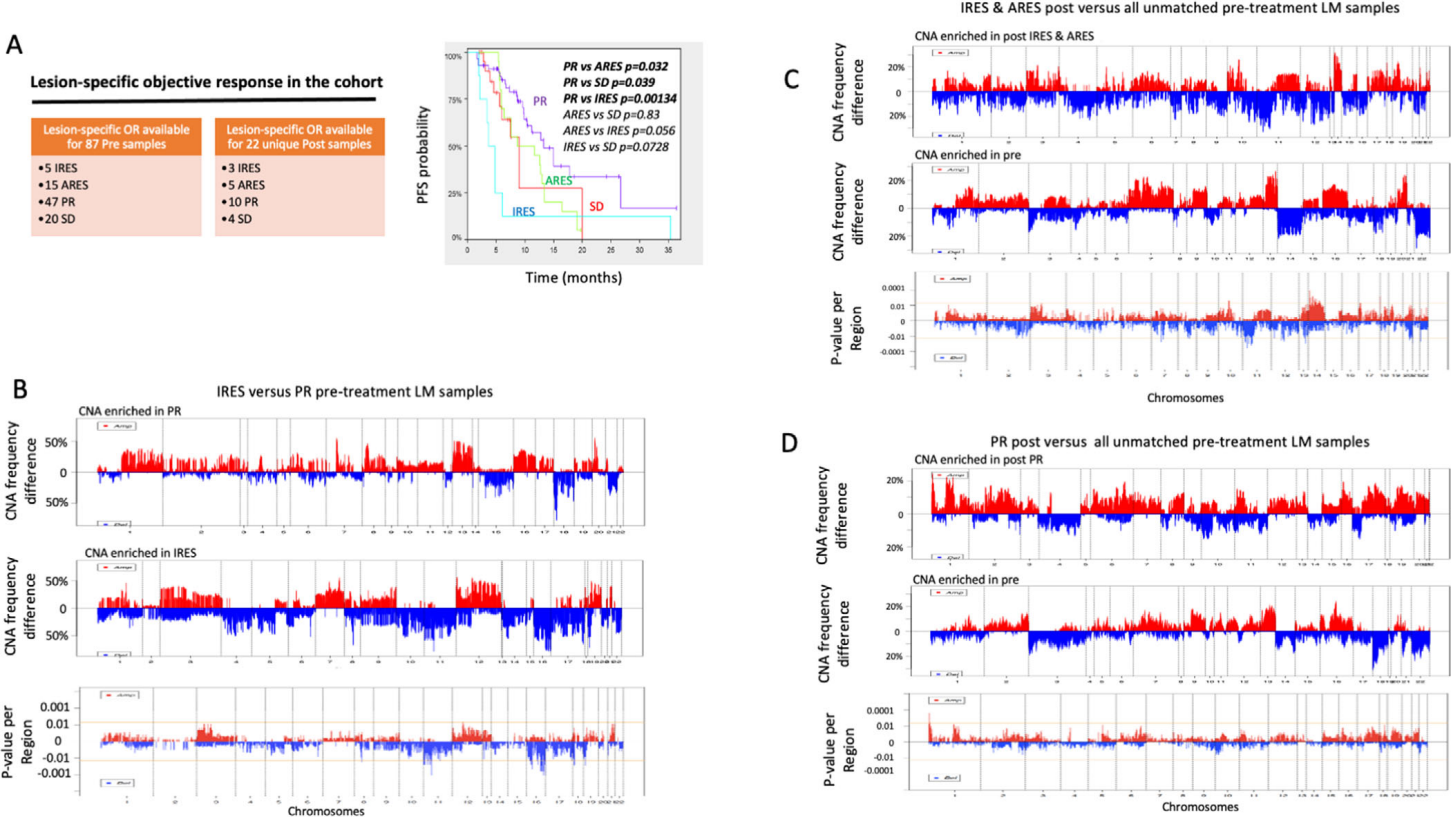
Copy number variation association with lesion specific objective response. (A) Lesion‐specific ORs were available for a subset of pre‐ and post‐samples allowing subsequent group comparisons and Kaplan‐Meier curves of patient PFS by lesion‐specific ORs. (B) CNA frequency differences (CNA enriched in IRES on the top panel; CNA enriched in PR samples on the middle panel) or *p* values of the frequency differences (lower panel) between IRES and PR pre‐samples. (C) CNA frequency differences (CNA enriched in post resistant on the top panel; CNA enriched in pre‐samples on the middle panel) or *p* values of the frequency differences (lower panel) between post IRES and ARES samples versus all unmatched pre‐treatment samples. (D) CNA frequency differences (CNA enriched in post PR on the top panel; CNA enriched in pre‐samples on the middle panel) or *p* values of the frequency differences (lower panel) between post PR samples and all pre‐treatment samples. Horizontal yellow line represents the significance threshold (*p* < 0.005). In all graphs, vertical dotted lines represent chromosomes boundaries. OR: objective response; Pre: pre‐treatment, Post: post‐treatment; LM: liver metastasis; PT: primary tumor; PR: partial responder; ARES: acquired resistance; IRES: intrinsic resistance; SD: stable disease

Lesion‐specific objective responses (OR) were assigned to a subset of samples (Figure 3A), given that “mixed response,” where one lesion might grow while another shrink in response to treatment, is more common than generally thought.^31^, ^32^, ^33^ We looked at both the lesion‐specific response and also overall patient's response and found that the lesion responses correlated with overall clinical outcomes based on PFS, with a significant difference between the lesions that were responding to treatment and each of the three other response groups (stable, intrinsic resistant, and acquired resistant) (Figure 3A). To identify CNA associated with therapeutic response, CNA profiles of lesions at baseline that responded to treatment (partial responder or PR lesions) were compared with the lesions that demonstrated intrinsic resistance (IRES lesions, no response), and CNA frequency differences were assessed (Figure 3B). While only one CN loss region was uniquely detected in PR samples, with a frequency of 76.6%, a larger number of CNAs were enriched in tumors that were intrinsically resistant (IRES lesions) (Table S6). Thirty‐two CN losses and one CN gain, harboring a total of 396 genes, showed higher frequencies in IRES lesions (63.5% in average) compared to lesions that responded to treatment (4.3% in average) (unadjusted *p* < 0.005; Figure 3B and Table S6). Among these genes, seven are classified as clinically actionable^29^ (*HRAS*, *WT1*, *PALB2*, *CBFB*, *CDH1*, *FANCA*, and *TUBB3*).

CNAs at resistance were investigated by comparing the profile of post‐samples that were or had become resistant to therapy (IRES and ARES ) to all unmatched pre‐samples (Figure 3C). Thirty‐five CN gains and 51 CN losses, harboring a total of 634 genes, were more frequently observed in post‐treatment resistant tumors (49.2% in average) compared to unmatched pre‐treatment samples (6.3% in average) (unadjusted *p* < 0.005; Figure 3C and Table S7A). One CN gain and one CN loss, harboring a total of five genes were more frequently found in pre‐treatment (85.6% and 56.7%, respectively) compared to post‐treatment resistant samples (37.5% and 0%, respectively; unadjusted *p* < 0.005, Table S7A). Overall, the expression of 54 genes were found significantly impacted by the genomic aberrations, including *SSTR1*, *LGALS3*, *RDH11*, *COQ6*, *PGF*, *RPS6KL1*, *KCNK10*, *CALM1*, *TDP1*, *LGMN*, *TYMS*, and *USP14*, all covered by CN gains enriched in post resistant‐samples, and classified as druggable according to the Drug‐Gene Interaction database (DGIdb).^29^ In contrast, the comparison of post‐treatment samples that were actually still responding to chemotherapy to all unmatched pre‐samples, only showed three CN gain regions, distinct from the regions previously identified (unadjusted *p* < 0.005, Figure 3D and Table S7B), and none of the genes present on these regions exhibited a dysregulation of the expression consistent with the CN aberration.

Within the cohort of the matched pairs, seven patients exhibited a chemotherapy resistant (five ARES and two IRES) post‐treatment lesion, which did not allow the identification of significant differences between post‐resistant and matched pre‐lesions. Nonetheless each pair was interrogated for the presence of the CNA identified in unmatched pairs analysis and revealed 57 aberrations (66.3%) consistently found present in the pre‐ or the post‐lesion in at least 1 of the matched pairs (Table S7A), which although small numbers, providing supporting evidence that these aberrations are to be found in resistant tumors post‐treatment.

Overall, our results revealed CNA variations after treatment that are specific to resistant lesions. These regions harbored genes already known in chemotherapy resistance, such as *TYMS*, but also genes that have not yet been described in this context that warrant further validation and investigation.

### 3.5 Copy number variation association with progression‐free survival

**FIGURE 4:**
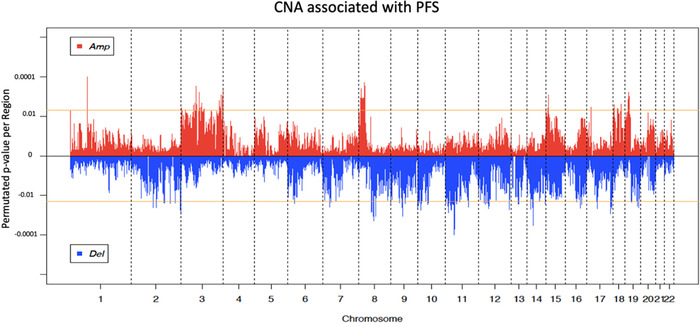
Copy number variation association with PFS. Permutated *p* value of CN gains (positive value) or CN losses (negative values) (*y*‐axis) derived from log‐rank tests are plotted as a function of chromosomal position (*x*‐axis). Horizontal yellow lines represent the significance threshold (permutated *p* < 0.005) and vertical dotted lines represent chromosomes boundaries.

We investigated whether novel CNAs associated with patient outcome could be identified in these metastatic samples, and whether these regions overlap with those previously reported in primary tumors associating PFS. Out of the 28,401 regions tested in pre‐treatment liver metastases, we identified 90 losses and 57 gains showing a significant association with PFS (log‐rank test, permutated *p* ≤ 0.005, Figure 4 and Table S8). Among the 1005 genes carried on these CNA regions, 61 (6.1%) showed a concordant and significant gene expression dysregulation in samples bearing the aberration (negative binomial test, FC > 1.5, FDR‐adjusted *p* < 0.1; Table S8).

**FIGURE 5:**
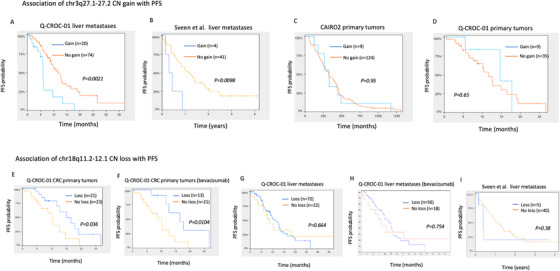
Kaplan‐Meier analyses for CN gains versus no gains of chr3q27.1‐q27.2 and CN loss versus no loss of chr18q11.2‐12 in Q‐CROC‐01, Sveen et al, and CAIRO2 cohorts. CN gain of chr3q27.1‐27.2 was tested for PFS association in (A) Q‐CROC‐01 liver metastasis (*n* = 94), (B) Sveen et al liver metastasis cohort (*n* = 45), (C) CAIRO2 primary tumor samples (*n* = 133), and (D) Q‐CROC‐01 CRC primary tumors (*n* = 44). CN loss of chr18q11.2‐12.1 association with a longer PFS was tested in (E) Q‐CROC‐01 CRC primary tumors (*n* = 44), (F) Q‐CROC‐01 CRC primary tumors from the sub‐group of patients treated with bevacizumab (*n* = 33), (G) Q‐CROC‐01 liver metastasis (*n* = 94), (H) Q‐CROC‐01 liver metastases from patients treated with bevacizumab (*n* = 74), and (I) Sveen et al liver metastases cohort (*n* = 45)

In order to validate the association with treatment response of these CNA regions, we interrogated the Sveen et al. cohort, which is the only publicly available cohort of metastatic CRC samples with clinical data from 45 patients.^19^ Nine CN losses and 9 CN gains (12.2%) were consistently and significantly associated with a shorter PFS in both our and the Sveen cohorts, including the CN gain covering *ETV5*, classified as clinically actionable in the DGIdb (Table S8). We interrogated our cohort of primary FFPE tumor samples to validate the aberrations identified in the liver metastatic sample cohort. This cohort displays a lower quality (robust variance quality score of 0.042 on average in liver metastases vs 0.086 on average in primary tumors), and thus CNAs consistently associated with PFS in both cohorts despite the technical differences would represent strong biomarker candidates. Six CNA regions (4.1%) were found associated with a shorter PFS in both cohorts. Among these regions, a CN loss spanned chr17q12 with six members of the chemokine CCL family (Table S8). The previously published CAIRO2 CRC sample cohort of 133 primary tumors was interrogated as well, and only 9 aberrations (6.1%) were overlapping with CNA regions identified in liver metastases. Two regions were shared by the two cohorts of primary tumors (Q‐CROC‐01 and CAIRO2), including the loss on chr17q12. No common CNA segments were found significantly associated with PFS in any the four cohorts of CRC, either of primary tumors and liver metastasis, which were interrogated. However, three CN losses, on chr16q21, chr17q25.3, and chr17q24.3‐q25.3, were found significantly associated with a shorter PFS in the two liver metastasis cohorts and in the CAIRO2 primary tumor cohort (Table S8). Fifteen CNAs (10.2%) were found significantly associated with a shorter PFS in both cohorts of metastatic samples, but not in the two primary tumor cohorts, which represents an important discovery that molecular characterization of metastases can provide additional genomic aberrations with prognostic information. Chr3q27.1‐q27.2 CN gain is one of these metastatic‐specific CNA candidates (Figures 5A‐5D) and is particularly interesting as this region harbors the *IGF2BP2*, *SENP2*, and *ETV5* genes, overexpressed in samples carrying the gain, that were previously reported to have a pro‐oncogenic role in many types of cancers.^34^, ^35^, ^36^

Overall, these analyses identified 147 CNA regions significantly associated with PFS in metastatic lesions of CRC patients. The association with PFS of 18 (12.2%) of these regions was validated in an independent cohort of metastatic samples, and the majority of these CNAs (15 out of 18) did not show association with PFS in the CAIRO2 cohort of primary tumors. While CNAs have been associated with patient outcome in the case of primary tumors (for review^37^), in the case of metastatic lesions, to our knowledge, these results provide the first evidence that new CNAs associated with patient outcome can be identified specifically in metastatic lesions, and this highlights the relevance of analyzing these lesions. Afterall, most new therapeutics are tested in metastatic patients, so understanding any specificities of this molecular context could be critical to evaluate these new agents, and potentially to better select patients for specific drug trials.

### 3.6 Copy number variation association with progression‐free survival in bevacizumab‐treated patients

As the majority (73%) of the patients in this study were treated with bevacizumab as part of their first‐line metastatic therapy (Table 1), we investigated whether CNAs that have shown strong association with PFS in the primary tumors of patients treated with bevacizumab are associated with outcome in the metastatic setting. CN loss of chr18q11.2‐q12.1 was previously reported to be associated with a prolonged PFS in three independent cohorts of primary tumors from mCRC patients treated with bevacizumab including the CAIRO2 cohort.^5^, ^38^ In our cohort of primary samples, we confirmed the association with a prolonged PFS (Kaplan‐Meier, *p* = 0.036), and observed a stronger association when only patients treated with bevacizumab were considered (Kaplan‐Meier, *p* = 0.0104) (Figures 5E and 5F). In contrast, we found that this loss is no longer associated with a prolonged PFS in metastatic samples. Indeed, we did not observe association in our liver metastasis samples, neither in the whole cohort (Kaplan‐Meier, *p* = 0.664), nor in the subset of patients who had been treated with bevacizumab (Kaplan‐Meier, *p* = 0.754) (Figures 5G and 5H). Furthermore, this loss was also found not to be associated with PFS in the Sveen et al cohort of liver metastases^19^ (*p* = 0.38) (Figure 5I). Thus, our data confirms that this aberration is only associated with clinical outcome in primary tumors, where bevacizumab is not in use, on the basis of large adjuvant trials of unselected patients.^39^, ^40^ This result therefore reinforces the critical importance of examining the tumor tissue that is actually being treated, which for bevacizumab is only in the metastatic setting, and not a more convenient surrogate (ie, primary tumor) in identifying and validating predictive biomarkers and therapeutic targets.

The entire CNA landscape of liver metasttic samples from patients treated with bevacizumab was also interrogated for association with PFS. Sixty‐six CNA segments harboring 61 genes, including *CDH1* and *SMARCA2*, showing a significant gene expression dysregulation in samples harboring the aberration were identified and listed in Figure S3B and Table S9. Since our cohort does not have adequate sample power to detect an association for the patients not receiving bevacizumab, at this point we present these candidates for further validation in a larger cohort.

### 3.7 Druggable candidate genes within functional CN gains enriched after or associated with chemotherapy treatment

To prioritize candidates for further validation studies or functional experiments, we selected genes categorised as *druggable genome* in DGIdb,^29^ within functional CN gain regions enriched in post‐treatment samples or in intrinsically resistant lesions in this study, or those found to be significantly associated with PFS in both cohorts of liver metastatic lesions which we and Sveen et al have interrogated. Thirty‐three genes met the selection criteria and are listed in Table 2. The association between CNA and gene expression of these candidates is shown in Figure S5. Of interest, the CN gain covering the gene *ETV5*, as well as its high level of expression, are associated with a shorter PFS in our cohort (Kaplan‐Meier, *p* = 0.03; Figure S6A). Furthermore, the association of mRNA levels with overall survival or relapse‐free survival was also confirmed in a publicly available dataset of rectum adenocarcinomas using Kaplan‐Meier Plotter online tool^41^ for *COQ6, GSTZ1, LGMN, RDH11, TGFB3*, *ETV5, PARP2, LGMN, TWSG1* and for *TSHR*, *SSTR1*, *CACNA1A*, *KCNK10*, respectively (Figures S6B‐N). Taken together our results revealed novel CN aberrations and genes, including *ETV5* as our top candidate, in liver metastatic lesions associated with clinical outcome in mCRC patients and highlights the need for profiling larger metastatic lesion cohorts to clarify the therapeutic and prognostic implication of each candidate.

**TABLE 2:** Druggable candidate genes within functional CN gains associated with chemotherapy treatment

Gene	CNA and clinical association* in Q‐CROC‐01 cohort (LM)1: gain in post‐sample vs pre‐samples2: gain in pos‐tresistant vs pre‐samples;3: gain associated with a shorter PFS in pre‐samples	Consistent mRNA regulation**	DGIdb classification	Drug‐gene interactions	Validation of CNA and clinical association in Sveen et al cohort***	High expression associated with a shorter PFS in Q‐CROC‐01 cohort (LM)	High expression associated with a shorter OS/RFS in rectum adenocarcinomas samples (KM plotter)^♣^, ^1^: OS in all stages^2^: OS in stage IV^3^: RFS in all stages
*ESR2*	1	Yes	Druggable genome, nuclear hormone receptor	Known	N/A	No	No
*GALC*	1	Yes	Druggable genome	Known	N/A	No	No
*GSTZ1*	1	Yes	Druggable genome	Known	N/A	No	**Yes**
*LAMA1*	1	Yes	Druggable genome	Known	N/A	No	No
*MLH3*	1	Yes	Druggable genome, DNA Repair	Unknown	N/A	No	No
*PARP2*	1	Yes	Druggable genome, DNA Repair	Known	N/A	No	**Yes**
*PSMC1*	1	Yes	Druggable genome, protease, transcription factor binding, tumor suppressor	Known	N/A	No	No
*RAD51B*	1	Yes	Druggable genome, clinically actionable, DNA Repair, tumor suppressor	Unknown	N/A	No	No
*SMAD2*	1	Yes	Clinically actionable, transcription factor complex and binding	Known	N/A	No	No
*SMCHD1*	1	Yes	Druggable genome	Unknown	N/A	No	No
*TGFB3*	1	Yes	Druggable, serine threonine Kinase, growth factor, cell surface	Known	N/A	No	**Yes**
*TSHR*	1	Yes	Druggable genome, clinically actionable, G protein couples receptor, cell surface	Known	N/A	No	**Yes**
*TWSG1*	1	Yes	Druggable genome	Unknown	N/A	No	**Yes**
*YES1*	1	Yes	Druggable genome, clinically actionable, tyrosine kinase	Known	N/A	No	No
*BCL2L2‐PABPN1*	1	Yes	Druggable genome	Unknown	N/A	No	No
*COQ6*	1, 2	Yes	Druggable genome	Unknown	N/A	No	**Yes**
*KCNK10*	1, 2	Yes	Druggable genome, transporter, ion chanel	Known	N/A	No	**Yes**
*PGF*	1, 2	Yes	Druggable genome, drug resistance, growth factor	Known	N/A	No	No
*TYMS*	1, 2	Yes	Druggable genome, drug resistance	Known	N/A	No	No
*USP14*	1, 2	Yes	Druggable genome, Protease, protease inhibitor, cell surface	Unknown	N/A	No	No
*CALM1*	2	Yes	Druggable genome, serine threonine kinase, transporter, protein phosphatase, ion chanel	Known	N/A	No	No
*LGMN*	2	Yes	Druggable genome, Protease	Unknown	N/A	No	**Yes** **^,^**
*RDH11*	2	Yes	Druggable genome, short chain dehydrogenase reductase	Known	N/A	No	**Yes**
*RPS6KL1*	2	Yes	Druggable genome, serine threonine kinase	Unknown	N/A	No	No
*SSTR1*	2	Yes	Druggable genome, G protein couples receptor	Known	N/A	No	**Yes**
*TDP1*	2	Yes	Druggable genome, DNA repair	Unknown	N/A	No	No
*LGALS3*	2	Yes	Druggable genome	Unknown	**Yes**	No	No
*ATP13A3*	3	Yes	Druggable genome, transporter, ABC transporter	Unknown	**Yes**	No	No
*CACNA1A*	3	Yes	Druggable genome, ion channel, transporter	Known	**Yes**	No	**Yes**
*ETV5*	3	Yes	Clinically actionable	Known	**Yes**	**Yes**	**Yes**
*LIPH*	3	Yes	Druggable genome, phospholipase, lipase	Unknown	**Yes**	No	No
*LMLN*	3	Yes	Druggable genome, protease, neutral zinc metallopeptidase	Unknown	**Yes**	No	No
*SENP2*	3	Yes	Druggable genome, protease	Unknown	**Yes**	No	No

* 1 and 2: two‐tailed Fisher's exact test, unadjusted *p* < 0.005; 3: log‐rank test, permutated *p* < 0.005.

** Consistent mRNA level and CN aberration; FDR‐corrected *p* < 0.1 (FigureS5).

*** Gain associated with shorter PFS in Sveen et al cohort (log‐rank test, permutated *p* < 0.05).

^♣^ Kaplan‐Meier Plotter database; *p* < 0.05.

^1^ 24 stage IV rectal adenocarcinomas interrogated for association between high gene expression and shorter overall survival (OS) time (Figures S6B‐F).

^2^ 165 all stages rectal adenocarcinomas interrogated for association between high gene expression and shorter OS (Figures S6G‐J).

^3^ 47 all stages rectal adenocarcinomas interrogated for association between high gene expression and shorter relapse‐free survival (RFS) time (Figures S6K‐N).

N/A, not applicable.

## 4 DISCUSSION

In this study, we explored the genomic landscape of CRC liver metastatic lesions at the copy number level, from baseline pre‐treatment to the appearance of clinical resistance, by collecting biospecimens and associated detailed clinical parameters. To the best of our knowledge, this represents the largest CRC liver metastatic sample set with associated treatment and outcome data profiled by next generation sequencing (WES), providing an unprecedented view of the aberrations acquired at baseline, over time of treatment, and at resistance. Larger mCRC sample collections have been analyzed by whole genome sequencing without specific reference to any therapeutic event or treatment response.^42^ The MSK‐impact cohort was profiled using a capture‐based NGS targeted panel of less than 500 genes, to characterize tumor mutations only.^43^

Our analysis of CNA landscapes between paired pre‐ and post‐treatment metastatic samples did not show significant divergences, perhaps because the sample size may still not be large enough. Obtaining serial biopsies from patients with metastatic disease is a monumental challenge, especially when the second biopsy is in the setting of disease progression and a change in therapy, which is likely why little of this sort of work has been done previously. In any case, the catalogue we are reporting here can serve in the future as a basis for studies with larger cohorts. Emerging technologies that facilitate collection of patient samples, such as cfDNA may facilitate this endeavor. While we acknowledge that an unmatched design has limitations, we believe it has important utility and relevance to identify frequent genomic variations induced by chemotherapy treatment that are common across different individuals with different genetic backgrounds. Our assumption is supported by the fact that our unmatched analysis identified a more frequent gain of *TYMS* in postsamples, that was previously reported in several independent studies to be amplified in CRC after 5‐FU treatment and also associated with a resistant phenotype, which was also captured by our analysis of unmatched samples. We identified one unique CN gain on chr18p11.32, containing the gene *TYMS*, that was significantly more frequently observed after treatment compared to baseline metastatic samples in unmatched pairs. Both amplification and overexpression of *TYMS* have been previously reported in primary tumors^44^ and liver metastases^45^ after 5‐Fluorouracil‐based chemotherapy, supporting the relevance of our cohort to study treatment response biology in mCRC. In addition to this region, we report additional CNA differentially enriched between the two groups, which warrant further validation in a larger sample set.

One particular strength of this study lies in the association of genomic changes with phenotypic features at the level of the lesion. Heterogeneous response to treatment in lesions within different metastatic sites or within the same organ has been reported in CRC and other types of cancers.^31^, ^32^, ^33^, ^46^ Since the homogeneity in phenotype is crucial in association studies, we used a RECIST‐based lesion specific OR to increase the quality and level of detail of phenotypic annotations. It provided an accurate insight into drug response at the level of the lesion and stratified the samples into phenotypic homogeneous subgroups. Despite the limitation resulting from generating smaller groups, this approach revealed CNAs at baseline enriched in intrinsically resistant tumors (IRES) compared to those that responded (PR), and we did identify CN differences after treatment in resistant lesions. As far as we know, this is the first attempt to associate focal CNA in CRC liver metastasis lesions with lesion OR and PFS, that resulted in the identification of promising candidates. Another potential confounding feature of this study is the fact that a small proportion of patients were treated with FOLFIRI (22%), not unlike many of the other studies cited, which might have complicated the investigation of the specific impact of each element of the backbone chemotherapy regimens on the CNA landscape. It may be also argued that in vitro validation of our candidate genes or regions should be present in this initial paper describing our work. Focusing on the characterization of the CNA landscape of metastases along with treatment response, our approach is novel as far as we know. We agree that ultimately these newly identified variants must be studied functionally, and at the protein level, ideally in a larger cohort that would provide sufficient power. Our efforts toward validation of our findings included both an orthogonal validation of CNA candidates using transcriptomic analysis on the same sample set and an external validation in an independent cohort of liver metastases as well as cross validation in a public database. Given the real‐world clinical challenges of collecting a large sample set of well‐annotated liver metastases at different times with respect to treatment, we feel it is an important accomplishment.

Extensive efforts to identify CNA of prognostic value of drug response in primary tumors have been previously made and led to the discovery of chr18q11.2‐q21.1 loss associated with a prolonged PFS in mCRC patients treated with bevacizumab.^5^, ^38^ In patients treated with bevacizumab in our cohort, this loss was significantly associated with a longer PFS only in primary tumor samples, which in fact is what has been demonstrated previously, but was no longer observed in liver metastatic samples, where this observation would have clinical meaning. This observation supports the notion that metastatic lesions are molecularly different from primary tumors and extrapolating one from the other for biomarker or therapeutic target identification may not be advised.

We reported that CN loss of *CDH1* and *SMARCA2* in liver metastatic samples were associated with PFS in the subset of patients treated with bevacizumab. Loss of *CDH1* (E‐Cadherin) was previously associated with invasiveness,^47^ disease progression,^48^ and shorter survival^49^ in CRC; however, the mechanisms of downregulation in CRC involved transcriptional and post‐transcriptional modifications.^50^, ^51^ The loss of chr 9p, the *SMARCA2* gene location, was associated with aggressiveness in clear cell renal carcinoma and is used as a prognostic marker.^52^, ^53^ Validation of these candidates in an independent liver metastasis cohort remains to be investigated since Sveen et al did not include patients treated with bevacizumab. The loss of *CDH1* being observed in a unique sample in the CAIRO2 cohort, association with patient outcome could not be confirmed in primary samples either. *SMARCA2* loss was observed in 12 samples in the CAIRO2 cohort but no significant association with PFS was observed (data not shown). The Sveen et al cohort of CRC liver metastases provided an important resource and allowed validation of certain regions associated with PFS, yet, with the limited sample size and treatment regimen being different (no bevacizumab), many candidates remain to be confirmed. In total, we selected 33 genes within the druggable genome category, targeted by a genomic aberration associated with drug response. Recent studies revealed the role of *ETV5*, our top candidate gene, in promoting CRC proliferation and angiogenesis in vitro and in vivo by inducing *VEGFA* expression, supporting *ETV5* as a prognostic biomarker and potential anti‐angiogenic therapeutic option in CRC.^54^, ^55^

Overall, the availability of this large amount of high‐quality global molecular data associated with detailed clinical parameters (available in Table S10) provides important biological data and will help future efforts in validating biomarkers in metastatic disease. Our study demonstrates that this collection of serial CRC metastatic samples during standard chemotherapy helped define important candidates by capturing the rapidly evolving CNA landscape. While the clinical feasibility of serial biopsies is not simple, such studies are critical to guide in the identification of genomic targets that could be more easily identified in liquid biopsies. Indeed, this systematic biospecimen collection, taken in a specific clinical context, has seeded a variety of studies with both technical and clinical implications,^56^, ^57^, ^58^, ^59^, ^60^ including emerging proteogenomic analyses.

## 5 CONCLUSION

By assessing copy number differences over time in metastatic lesions of patients with CRC, we identified alterations associated with therapeutic response. Orthogonal validation of expression changes and interrogation of independent cohorts revealed strong gene candidates, including *ETV5*. This study highlights the relevance of investigating metastases to identify novel genomic targets and biomarker candidates that are of likely clinical relevance for the treatment of metastatic disease, where most novel therapeutics are studied.

## ETHICS APPROVAL AND CONSENT TO PARTICIPATE

NCT00984048 and NCT01949194 were approved by the institutional review board at each participating hospital and complied with Good Clinical Practices, the principles of the Declaration of Helsinki, and all applicable regulatory requirements. Enrolled patients provided written informed consent.

## CONSENT FOR PUBLICATION

Not relevant.

6

## CONFLICT OF INTEREST

The authors declare no potential conflicts of interest.

## FUNDING

This work was supported by The Terry Fox Research Institute grant (102194) and the Fond de la recherche en santé du Québec (FRSQ) (101037) and Sanofi. This study was initiated by Q‐CROC and managed by Exactis Innovation, a Network of Centres of Excellence (NCE) funded organization.

## AUTHOR CONTRIBUTIONS

Conception and design: GB, CK, KG, MM. Provision of study materials and patients: GB, BS, BL, Y‐JK, RD, ES‐H, LS, FC, RB, MH, ST, PK, EC, AC. Histopathology review of tissues: AG. Lesion response evaluation: VP, MCT. Sample processing: AS, CH, ZD, KG. Collection and assembly of data: KG, MM, MCT, SM, CH, AS. Data analysis and interpretation: KG, MM, MCT, SM, CG, SH, GB, CK. Manuscript writing: KG, MM, GB, CK. Administrative support: Exactis Innovation. All authors read and approved the final manuscript.

## Supporting information

SUPPORTING INFORMATION

SUPPORTING INFORMATION

SUPPORTING INFORMATION

## Data Availability

The sequencing data are deposit at the  NCBI BioProject database and are accessible via the following link http://www.ncbi.nlm.nih.gov/bioproject/635121. The SNP array data are accessible via the following link:  https://www.ncbi.nlm.nih.gov/geo/query/acc.cgi?acc = GSE152178 Clinical parameters associated with each sample profiled are available in Table S10.

## References

[1] Howlader N , Noone AM , Krapcho M , et al. SEER Cancer Statistics Review, 1975–2016, National Cancer Institute. Bethesda, MD, https://seer.cancer.gov/csr/1975_2016/.

[2] Siegel RL , Miller KD , Jemal A . Cancer statistics, 2020. CA Cancer J Clin. 2020;70:7‐30. 31912902 10.3322/caac.21590

[3] Guinney J , Dienstmann R , Wang X , et al. The consensus molecular subtypes of colorectal cancer. Nat Med. 2015;21:1350‐1356. 26457759 10.1038/nm.3967 PMC4636487

[4] Postma C , Koopman M , Buffart TE , et al. DNA copy number profiles of primary tumors as predictors of response to chemotherapy in advanced colorectal cancer. Ann Oncol. 2009;20:1048‐1056. 19150955 10.1093/annonc/mdn738

[5] Haan JC , Labots M , Rausch C , et al. Genomic landscape of metastatic colorectal cancer. Nat Commun. 2014;5:5457. 25394515 10.1038/ncomms6457 PMC4243240

[6] Cancer Genome Atlas Network . Comprehensive molecular characterization of human colon and rectal cancer. Nature. 2012;487:330‐337. 22810696 10.1038/nature11252 PMC3401966

[7] Lievre A , Bachet JB , Boige V , et al. KRAS mutations as an independent prognostic factor in patients with advanced colorectal cancer treated with cetuximab. J Clin Oncol. 2008;26:374‐379. 18202412 10.1200/JCO.2007.12.5906

[8] Hu Z , Ding J , Ma Z , et al. Quantitative evidence for early metastatic seeding in colorectal cancer. Nat Genet. 2019;51:1113‐1122. 31209394 10.1038/s41588-019-0423-x PMC6982526

[9] Bhullar DS , Barriuso J , Mullamitha S , et al. Biomarker concordance between primary colorectal cancer and its metastases. EBioMedicine. 2019;40:363‐374. 30733075 10.1016/j.ebiom.2019.01.050 PMC6413540

[10] Vakiani E , Janakiraman M , Shen R , et al. Comparative genomic analysis of primary versus metastatic colorectal carcinomas. J Clin Oncol. 2012;30:2956‐2962. 22665543 10.1200/JCO.2011.38.2994 PMC3417049

[11] Brannon AR , Vakiani E , Sylvester BE , et al. Comparative sequencing analysis reveals high genomic concordance between matched primary and metastatic colorectal cancer lesions. Genome Biol. 2014;15:454. 25164765 10.1186/s13059-014-0454-7 PMC4189196

[12] Ishaque N , Abba ML , Hauser C , et al. Whole genome sequencing puts forward hypotheses on metastasis evolution and therapy in colorectal cancer. Nat Commun. 2018;9:4782. 30429477 10.1038/s41467-018-07041-z PMC6235880

[13] Narayan RR , Goldman DA , Gonen M , et al. Peripheral circulating tumor DNA detection predicts poor outcomes after liver resection for metastatic colorectal cancer. Ann Surg Oncol. 2019;26:1824‐1832. 30706231 10.1245/s10434-019-07201-5 PMC6511310

[14] Mamlouk S , Childs LH , Aust D , et al. DNA copy number changes define spatial patterns of heterogeneity in colorectal cancer. Nat Commun. 2017;8:14093. 28120820 10.1038/ncomms14093 PMC5288500

[15] Richman SD , Chambers P , Seymour MT , et al. Intra‐tumoral heterogeneity of KRAS and BRAF mutation status in patients with advanced colorectal cancer (aCRC) and cost‐effectiveness of multiple sample testing. Anal Cell Pathol. 2011;34:61‐66. 10.3233/ACP-2011-0005 PMC4605581 21483104

[16] Baldus SE , Schaefer KL , Engers R , et al. Prevalence and heterogeneity of KRAS, BRAF, and PIK3CA mutations in primary colorectal adenocarcinomas and their corresponding metastases. Clin Cancer Res. 2010;16:790‐799. 20103678 10.1158/1078-0432.CCR-09-2446

[17] Del Carmen S , Sayagues JM , Bengoechea O , et al. Spatio‐temporal tumor heterogeneity in metastatic CRC tumors: a mutational‐based approach. Oncotarget. 2018;9:34279‐34288. 30344942 10.18632/oncotarget.26081 PMC6188146

[18] Jeantet M , Tougeron D , Tachon G , et al. High intra‐and inter‐tumoral heterogeneity of RAS mutations in colorectal cancer. Int J Mol Sci. 2016;17:2015. 10.3390/ijms17122015 PMC5187815 27916952

[19] Sveen A , Loes IM , Alagaratnam S , et al. Intra‐patient inter‐metastatic genetic heterogeneity in colorectal cancer as a key determinant of survival after curative liver resection. PLoS Genet. 2016;12:e1006225. 27472274 10.1371/journal.pgen.1006225 PMC4966938

[20] Pitroda SP , Khodarev NN , Huang L , et al. Integrated molecular subtyping defines a curable oligometastatic state in colorectal liver metastasis. Nat Commun. 2018;9:1793. 29728604 10.1038/s41467-018-04278-6 PMC5935683

[21] Basik M , Aguilar‐Mahecha A , Rousseau C , et al. Biopsies: next‐generation biospecimens for tailoring therapy. Nat Rev Clin Oncol. 2013;10:437‐450. 23799370 10.1038/nrclinonc.2013.101

[22] Diaz Z , Aguilar‐Mahecha A , Paquet ER , et al. Next‐generation biobanking of metastases to enable multidimensional molecular profiling in personalized medicine. Mod Pathol. 2013;26:1413‐1424. 23743930 10.1038/modpathol.2013.81

[23] Therasse P , Arbuck SG , Eisenhauer EA , et al. New guidelines to evaluate the response to treatment in solid tumors. J Natl Cancer Inst. 2000;92:205‐216. 10655437 10.1093/jnci/92.3.205

[24] Beroukhim R , Getz G , Nghiemphu L , et al. Assessing the significance of chromosomal aberrations in cancer: methodology and application to glioma. Proc Natl Acad Sci U S A. 2007;;104:20007‐20012. 18077431 10.1073/pnas.0710052104 PMC2148413

[25] Peto R , Peto J . Asymptotically efficient rank invariant test procedures. J R Stat Soc. 1972;135:185‐207.

[26] Lee SY , Haq F , Kim D , et al. Comparative genomic analysis of primary and synchronous metastatic colorectal cancers. PLoS One. 2014;9:e90459. 24599305 10.1371/journal.pone.0090459 PMC3944022

[27] Astrup Jensen S , Vainer B , Janet Witton C , et al. Prognostic significance of numeric aberrations of genes for thymidylate synthase, thymidine phosphorylase and dihydrofolate reductase in colorectal cancer. Acta Oncol (Madr). 2008;47:1054‐1061. 10.1080/02841860801942158 18607850

[28] Rahman L , Voeller D , Rahman M , et al. Thymidylate synthase as an oncogene: a novel role for an essential DNA synthesis enzyme. Cancer Cell. 2004;5:341‐351. 15093541 10.1016/s1535-6108(04)00080-7

[29] Cotto AH , Feng YY , Kiwala S , et al. DGIdb 3.0: a redesign and expansion of the drug–gene interaction database. Nucleic Acids Res. 2017;46:D1068‐D1073. 10.1093/nar/gkx1143 PMC5888642 29156001

[30] Harada K , Okamoto W , Mimaki S , et al. Comparative sequence analysis of patient‐matched primary colorectal cancer, metastatic, and recurrent metastatic tumors after adjuvant FOLFOX chemotherapy. BMC Cancer. 2019;19:255. 30898102 10.1186/s12885-019-5479-6 PMC6429751

[31] Brunsell TH , Cengija V , Sveen A , et al. Heterogeneous radiological response to neoadjuvant therapy is associated with poor prognosis after resection of colorectal liver metastases. Eur J Surg Oncol. 2019;45:2340‐2346. 31350075 10.1016/j.ejso.2019.07.017

[32] Russo M , Siravegna G , Blaszkowsky LS , et al. Tumor heterogeneity and lesion‐specific response to targeted therapy in colorectal cancer. Cancer Discov. 2016;6:147‐153. 26644315 10.1158/2159-8290.CD-15-1283 PMC4744519

[33] Van Kessel CS , Samim M , Koopman M , et al. Radiological heterogeneity in response to chemotherapy is associated with poor survival in patients with colorectal liver metastases. Eur J Cancer. 2013;49:2486‐2493. 23692811 10.1016/j.ejca.2013.03.027

[34] Pickard A , McCance DJ . IGF‐binding protein 2—oncogene or tumor suppressor? Front Endocrinol (Lausanne). 2015;6:25. 25774149 10.3389/fendo.2015.00025 PMC4343188

[35] Oh S , Shin S , Janknecht R . ETV1, 4 and 5: an oncogenic subfamily of ETS transcription factors. Biochim Biophys Acta—Rev Cancer. 2012;1826:1‐12. 10.1016/j.bbcan.2012.02.002 PMC3362686 22425584

[36] Selby TL , Biel N , Varn M , et al. The Epstein‐Barr virus oncoprotein, LMP1, regulates the function of SENP2, a SUMO‐protease. Sci Rep. 2019;9:9523. 31266997 10.1038/s41598-019-45825-5 PMC6606635

[37] Ried T , Meijer GA , Harrison DJ , et al. The landscape of genomic copy number alterations in colorectal cancer and their consequences on gene expression levels and disease outcome. Mol Asp Med. 2019;69:48‐61. 10.1016/j.mam.2019.07.007 31365882

[38] van Dijk E , Biesma HD , Cordes M , et al. Loss of chromosome 18q11.2‐q12.1 is predictive for survival in patients with metastatic colorectal cancer treated with Bevacizumab. J Clin Oncol. 2018;36:2052‐2060. 29792754 10.1200/JCO.2017.77.1782

[39] André T , Vernerey D , Im SA , et al. Bevacizumab as adjuvant treatment of colon cancer: updated results from the S‐AVANT phase III study by the GERCOR Group. Ann Oncol. 2020;31:246‐256. 31959341 10.1016/j.annonc.2019.12.006

[40] de Gramont A , Van Cutsem E , Schmoll HJ , et al. Bevacizumab plus oxaliplatin‐based chemotherapy as adjuvant treatment for colon cancer (AVANT): a phase 3 randomised controlled trial. Lancet Oncol. 2012;13:1225‐1233. 23168362 10.1016/S1470-2045(12)70509-0

[41] Nagy A , Lanczky A , Menyhart O , Gyorffy B . Validation of miRNA prognostic power in hepatocellular carcinoma using expression data of independent datasets. Sci Rep. 2018;8:9227. 29907753 10.1038/s41598-018-27521-y PMC6003936

[42] Priestley P , Baber J , Lolkema MP , et al. Pan‐cancer whole‐genome analyses of metastatic solid tumours. Nature. 2019;575:210‐216. 31645765 10.1038/s41586-019-1689-y PMC6872491

[43] Yaeger R , Chatila WK , Lipsyc MD , et al. Clinical sequencing defines the genomic landscape of metastatic colorectal cancer. Cancer Cell. 2018;33:125‐136. 29316426 10.1016/j.ccell.2017.12.004 PMC5765991

[44] Varghese V , Magnani L , Harada‐Shoji N , et al. FOXM1 modulates 5‐FU resistance in colorectal cancer through regulating TYMS expression. Sci Rep. 2019;9:1505. 30728402 10.1038/s41598-018-38017-0 PMC6365533

[45] Watson RG , Muhale F , Thorne LB , et al. Amplification of thymidylate synthetase in metastatic colorectal cancer patients pretreated with 5‐fluorouracil‐based chemotherapy. Eur J Cancer. 2010;46:3358‐3364. 20727737 10.1016/j.ejca.2010.07.011 PMC2991554

[46] Menzies AM , Haydu LE , Carlino MS , et al. Inter‐ And intra‐patient heterogeneity of response and progression to targeted therapy in metastatic melanoma. PLoS One. 2014;9:e85004. 24400126 10.1371/journal.pone.0085004 PMC3882277

[47] Okugawa Y , Toiyama Y , Inoue Y , et al. Clinical significance of serum soluble E‐cadherin in colorectal carcinoma. J Surg Res. 2012;175:e67‐e73. 22277332 10.1016/j.jss.2011.11.009

[48] Gao M , Zhang X , Li D , et al. Expression analysis and clinical significance of eIF4E, VEGF‐C, E‐cadherin and MMP‐2 in colorectal adenocarcinoma. Oncotarget. 2016;7:85502‐85514. 27907907 10.18632/oncotarget.13453 PMC5356753

[49] Elzagheid A , Buhmeida A , Laato M , et al. Loss of E‐cadherin expression predicts disease recurrence and shorter survival in colorectal carcinoma. APMIS. 2012;120:539‐548. 22716209 10.1111/j.1600-0463.2011.02863.x

[50] Kitadai Y , Bucana CD , Ellis LM , et al. In situ mRNA hybridization technique for analysis of metastasis‐related genes in human colon carcinoma cells. Am J Pathol. 1995;147:1238‐1247. 7485388 PMC1869504

[51] Hu Y , Dai M , Zheng Y , et al. Epigenetic suppression of E‐cadherin expression by Snail2 during the metastasis of colorectal cancer. Clin Epigenetics. 2018;10:154. 30541610 10.1186/s13148-018-0592-y PMC6291922

[52] Jancewicz I , Siedlecki JA , Sarnowski TJ , Sarnowska E . BRM: the core ATPase subunit of SWI/SNF chromatin‐remodelling complex—a tumour suppressor or tumour‐promoting factor? Epig Chromatin. 2019;12:68. 10.1186/s13072-019-0315-4 PMC6852734 31722744

[53] La Rochelle J , Klatte T , Dastane A , et al. Chromosome 9p deletions identify an aggressive phenotype of clear cell renal cell carcinoma. Cancer. 2010;116:4696‐4702. 20629029 10.1002/cncr.25279

[54] Cheng X , Jin Z , Ji X , et al. ETS variant 5 promotes colorectal cancer angiogenesis by targeting platelet‐derived growth factor BB. Int J Cancer. 2019;145:179‐191. 30650178 10.1002/ijc.32071

[55] Feng H , Liu K , Ji X , et al. ETV5 promotes angiogenesis and accelerates bevacizumab resistance in colorectal cancer by transcriptionally activating VEGFA. SSRN Electron J. 2020. 10.1016/j.canlet.2018.09.026 30253191

[56] Alcaide M , Cheung M , Hillman J , et al. Evaluating the quantity, quality and size distribution of cell‐free DNA by multiplex droplet digital PCR. Sci Rep. 2020;10:12564. 32724107 10.1038/s41598-020-69432-x PMC7387491

[57] Alcaide M , Cheung M , Bushell K , et al. A novel multiplex droplet digital pcr assay to identify and quantify KRAS mutations in clinical specimens. J Mol Diagnostics. 2019;21:214‐227. 10.1016/j.jmoldx.2018.09.007 30472330

[58] Alcaide M , Yu S , Davidson J , et al. Targeted error‐suppressed quantification of circulating tumor DNA using semi‐degenerate barcoded adapters and biotinylated baits. Sci Rep. 2017;7:10574. 28874686 10.1038/s41598-017-10269-2 PMC5585219

[59] Blank‐Landeshammer B , Richard VR , Mitsa G , et al. Proteogenomics of colorectal cancer liver metastases: complementing precision oncology with phenotypic data. Cancers (Basel). 2019;11:1907. 10.3390/cancers11121907 PMC6966481 31805664

[60] Sinha S , Brown H , Tabak J , et al. Multiplexed real‐time polymerase chain reaction cell‐free DNA assay as a potential method to monitor stage IV colorectal cancer. Surgery (United States). 2019;166:534‐539. 10.1016/j.surg.2019.06.004 31378479

