# Supplementary material for: Copy number and transcriptome alterations associated with metastatic lesion response to treatment in colorectal cancer
Source: Clin Transl Med. 2021 May 1;11(4):e401. doi: 10.1002/ctm2.401 (PMC8087915; doi:10.1002/ctm2.401)
Supplement: Supplementary file 1 — SUPPORTING INFORMATION [file CTM2-11-e401-s002.docx]

Supplementary Method

**Tumor sample processing and quality assessment**

Snap frozen and washed RNALater samples were embedded in optimal cutting temperature (OCT) medium. Formalin samples were processed and embedded in paraffin by the Research Pathology Laboratory of the Jewish General Hospital. All samples were sectioned (4-5 um) and stained by Haematoxylin and Eosin (H&E).

All fresh frozen (FF) and RNA-later samples were embedded in optimal cutting temperature (OCT) medium as previously reported(1). Cryosections (5 μm thick) were cut and stained with hematoxylin and eosin (H&E). The percentage of normal cells, tumor cells and necrotic areas was determined by a pathologist and the threshold for suitable tumor cell area for extraction was set at ≥40% of the specimen including <20% of necrotic cells. When needed, biopsies were macro dissected to reach these thresholds. One representative sample per collection was selected for nucleic acid extraction. In the case of extraction or nucleic acid QC failure, the second sample was processed.

Concentration, purity and degradation of nucleic acid extracts were measured with the NanoDrop spectrophotometer (Thermo Fisher Scientific, Mississauga, ON, Canada). In addition, double stranded DNA concentration was measured using either the Qubit v2.0 Fluorometer (Thermo Fisher) or the PicoGreen dsDNA Assay kit (Thermo Fisher), while RNA integrity was assessed using the Agilent bioanalyzer 2100 (Agilent Technologies, Santa Clara, CA, USA). Samples with 260/280 absorbance ratio ≥ 1.8 and sufficient DNA quantity were used for WES profiling and samples with RIN≥3 were selected for total RNA sequencing.

**Objective response RECIST 1.0**

The Response Evaluation Criteria in Solid Tumours (RECIST(2)) is the widely accepted guideline to evaluate the total tumor burden by selecting up to five target lesions and calculating the average diameter change. This method only assesses the overall change in tumor burden and limits the observation of potential subtle differences in response among several metastases within a patient. With the objective to capture the change affecting each lesion during the treatment, the RECIST 1.0 was used as a guideline for evaluating LM lesion specific response. CT scan with contrast or MRI evaluations were scheduled at baseline, 8 weeks after treatment start, and every 12 weeks thereafter until treatment discontinuation. Each lesion that was biopsied and used for biomarker analysis was assessed as per the following criteria: a lesion was considered intrinsically resistant (IRES) if its longest diameter increased by ≥ 20% by the first 8-week evaluation; a lesion was categorized as partial response (PR) when a ≥ 30% decrease in the largest diameter since start of treatment was observed using the baseline measurement as reference; a lesion with a < 30% decrease in the longest diameter taking as reference the baseline measurement or a < 20% increase in the longest diameter taking as reference the smallest diameter was considered stable (SD); and a lesion with a PR or SD response followed by a ≥ 20% increase in the longest diameter taking as reference the smallest diameter was characterized as acquired resistant (ARES). When possible, matched pre- (at baseline) and post- (at progression) biopsies were collected from metastases in the same liver segment. Lesion response was measured on pre-biopsy samples in only 67 patients, matched pre and post-biopsies from 20 patients and post-biopsies from 21 patients. No more than one pre- and one post-biopsy samples was selected for analysis.

**Q-CROC-06 clinical trial**

NCT01949194 previously names Q-CROC-06 is a phase II exploratory study active at the same centres as Q-CROC-01 between September 2013 and December 2018. This study enrolled 48 patients with mCRC who had failed first-line therapy with an oxaliplatin-containing regimen with or without bevacizumab (Figure 1) and required a metastatic tumor biopsy in order to determine the efficacy of a particular second-line treatment. Since these patients, and their tumors have been exposed to and were resistant to the very same treatments, 19 liver metastatic samples meeting the same quality criteria set for the Q-CROC-01 study samples, were grouped with the post-samples of the Q-CROC-01 study in subsequent molecular analyses. However, the information relative to the PFS of the first-line therapy for these patients was not collected.

**CytoScan HD profiling and data analysis**

Whole genome SNP analysis was performed on 45 selected LM samples at Genome Quebec Innovation Centre using Affymetrix®CytoScan™ high density (HD) SNP-array (Affymetrix) following manufacturer instructions. Briefly, genomic DNA (gDNA) was re-quantified at Genome Quebec Innovation Centre using a NanoDrop Spectrophotometer ND-1000 (NanoDrop Technologies, Inc.) and its approximating average size was assessed using a 1% agarose gel (E-Gel 48-well, Life Technologies) with High Range DNA Marker (Life Technologies). Two hundred fifty ng of gDNA from each tumor sample was first digested with Nsp I restriction endonuclease and ligated to specific adaptors that recognize the cohesive four base pair overhangs. The digested and ligated sample was then amplified by PCR using Titanium Tag Polymerase (CloneTech). Each PCR reaction was run on a 2% agarose gel (E-Gel 48-well, Life Technologies) with PCR Marker (Affymetrix) to ensure consistent PCR products distribution between 150 bp to 2000 bp. The PCR amplification was followed by a purification step with Purification bead. Purified PCR product was quantified by Nanodrop in order to assess the average DNA yield. PCR products were fragmented according to specific Affymetrix recommendation (Technical Note: P/N 703289 Rev1). Fragmentation size distribution was confirmed by majority of distribution between 25 to 125 bp, using a 4% agarose gel (E-Gel 48-well, Life Technologies) with TrackIt 25 bp DNA Marker (Life Technologies). After labeling with biotin, the DNA target was hybridized to the CytoScan® HD array (Affymetrix) and incubated at 49^0^C in the Genechip® Hybridization oven 640 (Affymetrix) for 17 hours at 60 rpm. CytoScan array were then washed in a GeneChips® Fluidics Station 450 (Affymetrix). Microarrays were finally scanned on a GeneChip® scanner 3000 (Affymetrix). The raw .CEL files were processed using Nexus Copy Number ^TM^ (BioDiscovery Inc, CA, USA) using SNP-FASST2 Segmentation Algorithm in which B-allele frequency probes are assigned to a range of possible states and a combination of the BAF and Log-R states are used to make the final copy number and allelic event calls. Quadratic correction and median probes re-centering were applied as well as removal of 3% outliers. The significance threshold for segmentation was set at 5.10^-7^ also requiring a minimum of 20 probes per segment and a maximum probe spacing of 1000 between adjacent probes before breaking a segment. The log ratio thresholds for single copy gain and single copy loss were set at 0.1 and -0.15, respectively. The log ratio thresholds for two or more copy gain and homozygous loss were set at 0.7 and -1.1 respectively. The Homozygous Frequency Threshold was set to 0.85. The Homozygous Value Threshold was set to 0.8. Sex chromosomes were excluded from the analysis. Robust variance sample quality calculation was below 0.15 for all the samples analysed.

**Copy number aberration analysis of external datasets (GSE63490, GSE36864 and GSE53799) using Nexus Copy Number and correlation analysis**

To validate our CNA calling methodology using Nexus Copy Number software, we downloaded the raw CNA data from 3 independent datasets available on NCBI GEO (GSE63490, GSE36864 and GSE53799), analyzed the CNA profile using Nexus Copy Number software and compared the results obtained with available published results. GSE63490 dataset was composed of 134 LM deposits from 45 CRC patients, profiled by Affymetrix Genome-Wide Human SNP 6.0 Array(3). In Nexus the corresponding 134 CEL. Files were processed using the SNP-FASST2 Segmentation Algorithm with the pre-set default settings (quadratic correction, median recenter probes and removal of 3% outliers). A minimum of 20 probes per segment was required. A list of regions of CNA was computed using the aggregate tool of Nexus (peaks only), which was then used to compare frequency of the CNA regions listed in the S3 Text Table A in Sveen et al.(3) publication.

The GSE36864 dataset was composed of 349 PT samples from 349 mCRC patients profiled using Agilent SurePrint G3 CGH array (4X180K) CCA_VUMC design AMADID#022522(4). All 349 text files were uploaded in Nexus and processed using FASST2 Segmentation algorithm with pre-set default settings (quadratic correction, median recenter probes, minimum of 3 probes per segments and removal of 3% outliers). Quadratic systematic GC wave correction was applied using custom correction file provided by BioDiscovery and the probe coordinates were remapped from hg18 to hg19. A list of CNA was obtained with the aggregate tool in Nexus (peaks only) and the frequencies of overlapping regions listed in the supplementary table 2 and 3 of the Haan et al.(4) publication were compared.

From the last dataset, GSE53799, we selected the 15 LM samples from 15 CRC patients profiled using Affymetrix CytoScan HD Array (Lee et al.(5)). The 15 Affymetrix CEL files were loaded along with their 15 matched normal CEL files for paired analysis in Nexus and were processed using SNP-FASST2 Segmentation algorithm with default settings (quadratic correction, median recenter probes and removal of 3% outliers). A minimum of 20 probes per segment was required. Since only the number of aberrations were available from Lee et al.(5), the number of CNA found in our analysis using the aggregate tool (peaks only) in Nexus was compared with those reported in Lee et al. (Table 2). For all the comparisons using the three external datasets, the coefficient of determination (R^2^) was computed from regression analyses to evaluate the results reproducibility using Nexus (Supplementary Fig.2). Our analysis showed 72%, 95% and 98% of correlation with CNA calls reported in Sveen et al.(3), Lee et al.(5), and Haan et al.(4), respectively (Supplementary Fig. 1A-C).

**Copy number aberration analysis of external datasets (GSE63490 and GSE36864) and association with progression free survival**

To identify putative association between CNA and PFS in the 2 external datasets, the log-rank statistic in Nexus was used on 45 LM samples from 45 CRC patients in GSE63490 and on 133 PT samples from 133 mCRC patients in CAIRO2 arm-A in GSE36864. Minimal region length was 1 Kb and minimum number of patients per group was 3. The p-value is calculated by permuting the progression-free survival time for each sample and comparing the log-rank statistic for the permuted data to the original data. The threshold used was p-value ≤ 0.05. To compare survival times between 2 groups, Kaplan-Meier curves were generated, and p-values were computed using the log-rank test.

**Whole Exome Sequencing (WES)**

In total, 204 tumor DNA and 120 normal DNA samples were profiled by WES in 5 batches. The following table summarizes the sequencing strategies used for each batch.

The library capture of the exome was performed using SureSelect XT Human All Exon V5 or V6 + UTR (Agilent, USA). Sequencing was performed on the Illumina HiSeq 2500 or 4000 platform to reach an average number of reads per sample in the range of 46.6 to 62.2 million.

| **Batch** | **Number of DNA samples** | **Sample types** | **Input (ng)** | **Library Capture (Agilent)** | **Sequencing platform**  **(paired-end=100)** | **Median depth of coverage** |
| --- | --- | --- | --- | --- | --- | --- |
| 1 | 51 | 32 LM and 19 normal | 500 | SureSelect Exome V5+UTR | Illumina HiSeq 2500 (PE100) | 103x |
| 2 | 90 | 50 LM and 40 normal | 200 | SureSelect Exome V6+UTR | Illumina HiSeq 2500 (PE 125) | 138x |
| 3 | 98 | 56 LM and 42 normal | 200 | SureSelect Exome V6+UTR | Illumina HiSeq 4000 (PE 100) | 143x |
| 4 | 47 | 47 PT | 50 | SureSelect Exome V6+UTR | Illumina HiSeq 4000 (PE 100) | 188x |
| 5 | 38 | 19 LM and 19 normal | 200 | SureSelect Exome V6+UTR | Illumina HiSeq 4000 (PE 100) | 154x |

Reads were trimmed using Trimmomatic (v0.35)(6), removing adaptor, first four bases from the start of each read, and low-quality bases at the end of each read using a 4bp sliding window to trim where average window quality fell under 30. Trimmed reads ≤ 30 bp were discarded. The clean reads were then aligned to the reference genome hg19 (GRCh37) using BWA-MEM v0.7.13(7) using –M parameter. Duplicated reads were marked and filter out such as only unique DNA fragments were used in the subsequent analysis using Picard v2.1.0 (<http://broadinstitute.github.io/picard/>).

**Copy Number Aberration Analysis from WES**

BAM files were imported into Nexus Copy Number^TM^ software for CNA analysis using matched normal DNA from blood samples when available. B-Allele Frequency parameters were set to reject reads with read depth less than 20 and MAPQ less than 30. Bases with base quality less than 20 were excluded. The significance threshold for segmentation was set at 1.0E^-6^ also requiring a minimum of 5 probes per segment and a maximum probe spacing of 1000 Kbp between adjacent probes before breaking a segment. The log ratio thresholds for single copy gain and single copy loss were set at 0.18 and -0.18, respectively. The log ratio thresholds for two or more copy gain and homozygous loss were set at 0.6 and -1.0 respectively. Median recenter probes were applied as well as removal of 3% outliers. Quadratic Correction was applied where the bias values, including percent GC content and fragment length, are used to create a quadratic model whose parameters are estimated using the least squares method. The estimate is then subtracted from the probe Log2Ratio to obtain the corrected probe values. The robust variance sample quality score threshold was ≤ 0.09 and ≤ 0.35 for LM and PT samples respectively. Based on these criteria, seven post-treatment LM samples, all from the same patient were excluded from further CNA analyses.

**Copy Number Aberration calling comparison between WES and SNP-array**

A CNA call comparison was performed on the 45 samples from 35 patients that were profiled using both WES and CytoScan HD array technologies. CNA analysis from WES and from CytoScan analysis were performed as described in *DNA Copy Number Data Analysis from Whole Exome Sequencing* section of the main material and method and the *CytoScan HD profiling and data analysis* section of the supplemental material and methods respectively. In both analyses, a list of CNA regions was computed using the aggregate tool (peaks only) in Nexus, and only the regions with p-value ≤ 0.05 were used for the following analysis. The percentage of genomic change was calculated by adding up the length of all the gains and losses and by dividing the resulting number by the human genome size. To compare the two technologies at the gene level, the comparison tool of Nexus Copy Number was used. Genes contained in significant regions (p≤0.01) with a difference of 25% between both technologies were called as enriched in one of the technologies. The correlation in CNA frequency assessment between both technologies was estimated by intersecting the aggregate files using bedtools (Quinlan, AR 2010-841). Within a same type of event, 2 intervals were considered overlapping if they had at least 50% of reciprocal overlap.

CNA frequency plots derived from both platforms were highly similar and the degree of correlation reached 61 and 90 % for CN losses and CN gains respectively (Supplementary Fig. 2A and G). However, due to the coverage difference (exome versus genome), the total number of events was drastically lower in WES compare to SNP-array analysis while the distribution of CNA gains and losses remained comparable (Supplemental figure 2B and C). The length of the CNA events identified was greater in WES compared to SNP-array (Supplemental figure 2F) and yet, overall, the percent of genome changed remained highly similar (30% in SNP-array versus 26% in WES, Supplemental figure 2D) and 98.7% of the refseq genes showed identical CNA calls in either WES and SNP (Supplemental figure 2E).

**Validation of CNA regions associated with progression-free survival in independent datasets**

To validate the prognostic value of CNA regions identified in Q-CROC-01 cohort, we performed CNA analysis on external raw datasets available on the NCBI GEO that have PFS data available: GSE63490(3) and GSE36864(4). From the GSE63490 cohort, we selected 45 LM samples from 45 CRC patients (1 lesion per patient, with the highest quality value) and performed CNA analysis using Nexus as described in Data Supplement. From the GSE36864 cohort, we performed analysis on data from 133 PT samples on the CAIRO2 arm-A cohort of patients, as the first-line treatment of this group of patients (capecitabine, oxaliplatin and bevacizumab) was closest to the one of our cohort.

The survival predictive power in both datasets was evaluated using Log-rank test statistic as described in the Supplementary Method. Significant regions were then intersected with the CNA regions significantly associated with PFS in our cohort using Bedtools and two intervals were considered overlapping if they had at least 1000 bp and 20% of reciprocal overlap and were the same type of events.

**RNA sequencing**

In total, 135 RNA samples were sequenced in 4 batches. All samples showed a RIN>3. For each batch, the number of samples, the information relative to RNA sequencing strategy and the mean coverage per sample was reported in the table below.

| **Batch** | **Number of samples** | **Sample types** | **Input (ng)** | **Library Type** | **Sequencing platform** | **Average number of reads per sample (millions of reads)** |
| --- | --- | --- | --- | --- | --- | --- |
| 1 | 21 | 21 LM | 500 | Stranded mRNA | Illumina HiSeq 2500 (PE 75) | 57.2 |
| 2 | 44 | 44 LM | 200 | rRNA-depleted stranded (HMR) | Illumina HiSeq 2500 (PE 100) | 70 |
| 3 | 51 | 51 LM | 200 | rRNA-depleted stranded (HMR) | Illumina HiSeq 4000 (PE 100) | 69.1 |
| 4 | 19 | 19 LM | 250 | rRNA-depleted stranded (HMR) | Illumina HiSeq 4000 (PE 100) | 75.4 |

Reads were trimmed using Trimmomatic (v0.35) (6), removing adaptor, first four bases from the start of each read, and low-quality bases at the end of each read using a 4bp sliding window to trim where average window quality fell under 30. Trimmed reads < 30 bp were discarded. Clean reads were then aligned to the reference genome hg19 (GRCh37) using STAR v2.3.0(8). Gene counts were obtained using featureCount(9) with custom gtf files. Counts were normalized using the library size.

**Kaplan Meier plot using expression data**

Association between progression free survival and RNAseq data was assessed by splitting the cohort based on the median expression of the gene tested into two groups: High and Low. Kaplan Meier curves were generated using the “*survival*” R package, and difference between the two groups was tested using the *survdiff* function from the same package.

**Final tables preparation**

Adjacent regions with same event type were concatenated in the final tables. Numerical fields were added (Region Length) or the average between all the regions combined was computed (p-value, difference, frequency in post, frequency in pre, PFS). Characters or string fields (Gene Symbol, miRNA and Concordant Genes) are the concatenation of the unique string present in the regions combined for these fields.

**Classical Cosmic genes CNA frequency comparison between pre- and post-samples**

A list of the classical cosmic genes was downloaded from <https://cancer.sanger.ac.uk/cosmic> and used to query CN loss and gain events targeting each gene using the query tool (option completely covered) of Nexus Copy Number software in our cohort of samples. The significance of frequency difference between the pre and post samples was assessed using two-tailed Fisher’s exact test with a multiple testing correction p-value cut-off of 0.05.

**Statistics**

A Bonferroni threshold for the number of CNAs tested was determine for each comparison to control our family-wise error rate at 5%. The sample size needed for a 30% difference in the rate of observing a CNA and an 80% power was determined and listed in the table below.

| Groupe comparison | Number of CNA tested | Bonferroni threshold | Sample size needed |
| --- | --- | --- | --- |
| Unmatched pre versus post | 29,736 | 1.7E-6 | 259 |
| Matched pair pre versus post | 13,652 | 3.76E-6 | 244 |
| IRES versus PR pre samples | 12,505 | 4.1E-6 | 504 |
| Post ARES & IRES versus unmatched post | 22,860 | 2.24E-6 | 526 |
| Post PR versus unmatched post | 28,401 | 2.48 E-6 | 438 |

**Supplementary Figure legends:**

**Supplementary Figure 1: Sample flowchart.** Dataset of DNA copy number and RNAseq profiles based on quality control and selection criteria.

**Supplementary Figure 2: Validation of CNA inference workflow using Nexus Copy Number and CNA calling comparison between WES and SNP-array platform using 45 DNA samples.** (A) Two independent SNP-array datasets of CRC LM samples (Sveen et al. and Lee et al.) and one CGH array dataset of CRC PT samples (Haan et al.) have been downloaded and analyzed using Nexus Copy Number. The frequencies of the number of CNA segments identified have been compared to those reported in each manuscript and a correlation coefficient was inferred in each case. In all graphs, x-axis represent the results obtained using Nexus Copy Number and the y-axis the results reported in the corresponding publications. (B) CNA frequency plots of 45 mCRC samples profiled with CytoScan HD and WES platforms and analyzed using Nexus Copy Number. On both plots, Y axis shows frequency of gains (positive values, blue) and losses (negative values, red) and are shown as a function of chromosome region (x axis). Bar plots representing and comparing the number of events (C), the proportion of each type of events (D), the percentage of genomic changes (E) and the percentage of genes enriched (F) in the same 45 samples for each technology. (G) Size distribution of each event types using both technologies. Dashed lines represent the mean length for the event type. (H) Scatter plots comparing event frequencies identified using CytoScan HD and WES platforms. Linear model is shown as a red line.

**Supplementary Figure 3: Aggregates copy number aberrations of 119 liver metastastic samples and association analysis with PFS in 74 bevacizumab patients.**

(A) Aggregate plot displays copy number log ratio of 119 LM samples from 119 patients. The plot above the 0 line is for the gain aggregates and that below 0 is for the loss aggregates. The aggregates are scaled to 1/# of samples so that if all samples participate, the value corresponds to the average value at that location. The gray shading represents the significant GISTIC Regions.

(B) Permutated p-value associated with CN gains (positive value, red) or CN losses (negative values, blue) (y-axis) derived from log-rank tests performed using the PFS data collected from 74 patients treated bevacizumab are plotted as a function of chromosomal position (x-axis). Horizontal yellow lines represent the significance threshold (permutated p-value < 0.005) and vertical dotted lines represent chromosomes boundaries.

**Supplementary Figure 4: Venn diagrams illustrating intersections of gene lists in CN events identified using GISTIC in Q-CROC-01, CAIRO2 and TCGA cohorts.**

**(**A): Genes in CN gains. (B) Genes in CN losses.

**Supplementary Figure 5: Correspondence between copy number change and gene expression in selected candidates identified in group comparison and association analyses.** Box plots inferred from RNAseq data showing the impact of copy number changes on the expression (count value) of selected genes lying in CN aberrations found significantly different in frequency between post and pre-treatment samples (A), in post-resistant versus pre-treatment samples (B) and in the association study between CNA and PFS (C). p: FDR adjusted p-value. *Expression values for samples with CN gain are shown in the Figure 2. **Candidate among the 231 cosmic genes.

**Supplementary Figure 6: Kaplan Meier analyses of survival for high expression versus low expression of selected gene candidates in our QCROC-01 cohort and in Kaplan Meier Plotter database of adenocarcinomas.**

(A) High expression versus low expression (based on the median) of *ETV5* was tested for their association with PFS in Q-CROC-01 liver metastasis cohort. (B-F) Association between OS and gene expression level of *COQ6, GSTZ1, LGMN, RDH11 and TGFB3* was shown by Kaplan Meier analysis in 24 stage IV rectum adenocarcinomas. (G-J) Association between OS and gene expression level of *ETV5, PARP2, LGMN, and TWSG1* was shown by Kaplan Meier analysis in 165 rectum adenocarcinomas (all stages). (K-N) Association between RFS and gene expression level of *TSHR*, *SSTR1*, *CACNA1A* and *KCNK10* was shown by Kaplan Meier analysis in 47 rectum adenocarcinomas (all stages).

Reference

1. Diaz Z, Aguilar-Mahecha A, Paquet ER, Basik M, Orain M, Camlioglu E, et al. Next-generation biobanking of metastases to enable multidimensional molecular profiling in personalized medicine. Mod Pathol [Internet]. 2013/06/08. 2013;26:1413–24. Available from: https://www.ncbi.nlm.nih.gov/pubmed/23743930

2. Therasse P, Arbuck SG, Eisenhauer EA, Wanders J, Kaplan RS, Rubinstein L, et al. New guidelines to evaluate the response to treatment in solid tumors. J Natl Cancer Inst. 2000;

3. Sveen A, Loes IM, Alagaratnam S, Nilsen G, Holand M, Lingjaerde OC, et al. Intra-patient Inter-metastatic Genetic Heterogeneity in Colorectal Cancer as a Key Determinant of Survival after Curative Liver Resection. PLoS Genet [Internet]. 2016/07/30. 2016;12:e1006225. Available from: https://www.ncbi.nlm.nih.gov/pubmed/27472274

4. Haan JC, Labots M, Rausch C, Koopman M, Tol J, Mekenkamp LJ, et al. Genomic landscape of metastatic colorectal cancer. Nat Commun [Internet]. 2014/11/15. 2014;5:5457. Available from: https://www.ncbi.nlm.nih.gov/pubmed/25394515

5. Lee SY, Haq F, Kim D, Jun C, Jo HJ, Ahn SM, et al. Comparative genomic analysis of primary and synchronous metastatic colorectal cancers. PLoS One [Internet]. 2014/03/07. 2014;9:e90459. Available from: https://www.ncbi.nlm.nih.gov/pubmed/24599305

6. Bolger AM, Lohse M, Usadel B. Trimmomatic: a flexible trimmer for Illumina sequence data. Bioinformatics [Internet]. 2014/04/04. 2014;30:2114–20. Available from: https://www.ncbi.nlm.nih.gov/pubmed/24695404

7. Li H, Durbin R. Fast and accurate short read alignment with Burrows-Wheeler transform. Bioinformatics [Internet]. 2009/05/20. 2009;25:1754–60. Available from: https://www.ncbi.nlm.nih.gov/pubmed/19451168

8. Dobin A, Davis CA, Schlesinger F, Drenkow J, Zaleski C, Jha S, et al. STAR: ultrafast universal RNA-seq aligner. Bioinformatics [Internet]. 2012/10/30. 2013;29:15–21. Available from: https://www.ncbi.nlm.nih.gov/pubmed/23104886

9. Liao Y, Smyth GK, Shi W. featureCounts: an efficient general purpose program for assigning sequence reads to genomic features. Bioinformatics [Internet]. 2013/11/15. 2014;30:923–30. Available from: https://www.ncbi.nlm.nih.gov/pubmed/24227677
