# Supplementary material for: Copy number and transcriptome alterations associated with metastatic lesion response to treatment in colorectal cancer
Source: Clin Transl Med. 2021 May 1;11(4):e401. doi: 10.1002/ctm2.401 (PMC8087915; doi:10.1002/ctm2.401)
Supplement: Supplementary file 2 — SUPPORTING INFORMATION [file CTM2-11-e401-s001.pdf]

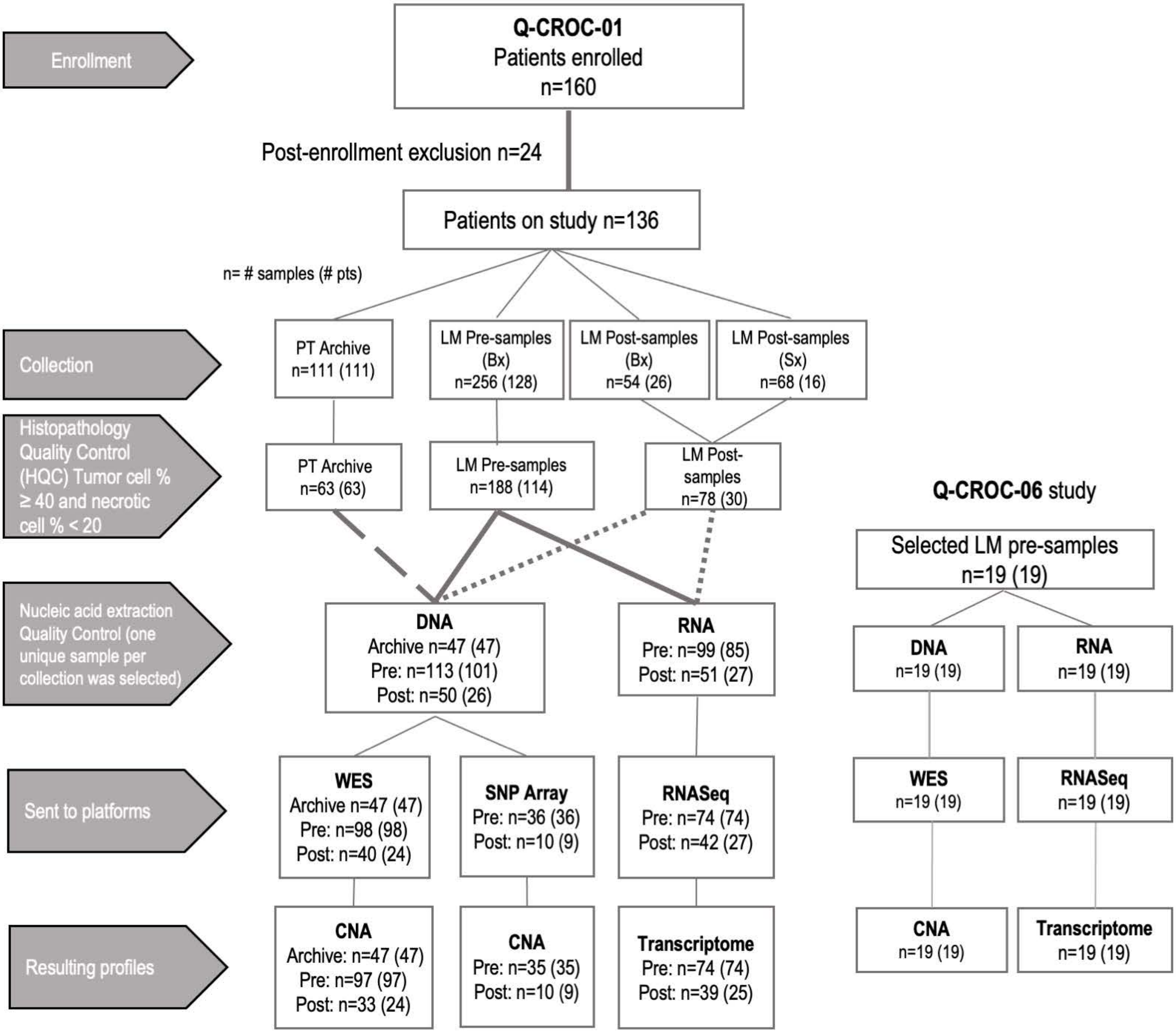

Supplementary Figure 1: Sample flowchart. Dataset of DNA copy number and RNAseq profiles based on quality control and selection criteria.

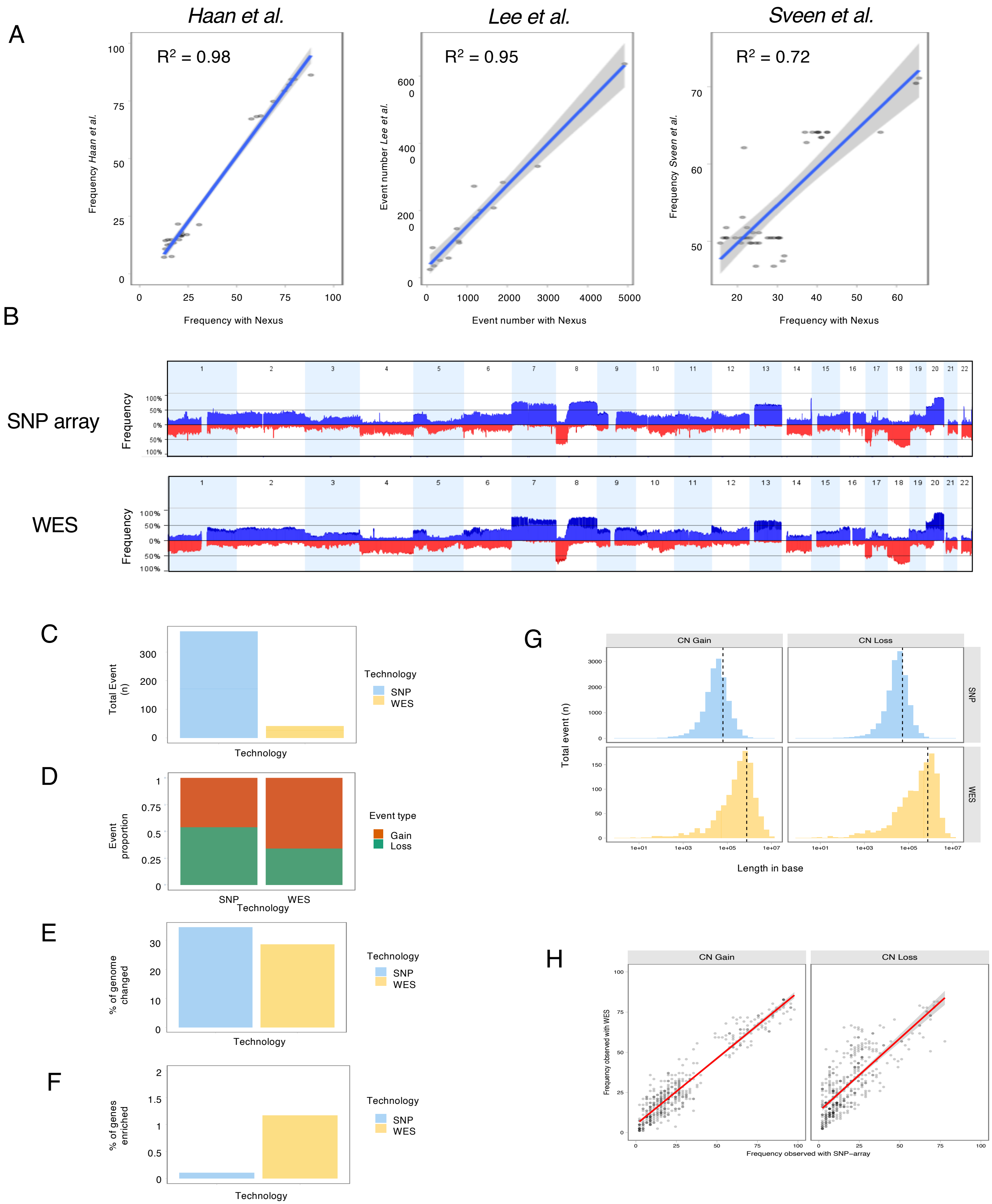

Supplementary Figure 2: Validation of CNA inference workflow using Nexus Copy Number and CNA calling comparison between WES and SNP-array platform using 45 DNA samples. (A) Two independent SNP-array datasets of CRC LM samples (Sveen et al. and Lee et al.) and one CGH array dataset of CRC PT samples (Haan et al.) have been downloaded and analyzed using Nexus Copy Number. The frequencies of the number of CNA segments identified have been compared to those reported in each manuscript and a correlation coefficient was inferred in each case. In all graphs, x-axis represent the results obtained using Nexus Copy Number and the y-axis the results reported in the corresponding publications. (B) CNA frequency plots of 45 mCRC samples profiled with CytoScan HD and WES platforms and analyzed using Nexus Copy Number. On both plots, Y axis shows frequency of gains (positive values, blue) and losses (negative values, red) and are shown as a function of chromosome region (x axis). Bar plots representing and comparing the number of events (C), the proportion of each type of events (D), the percentage of genomic changes (E) and the percentage of genes enriched (F) in the same 45 samples for each technology. (G) Size distribution of each event types using both technologies. Dashed lines represent the mean length for the event type. (H) Scatter plots comparing event frequencies identified using CytoScan HD and WES platforms. Linear model is shown as a red line.

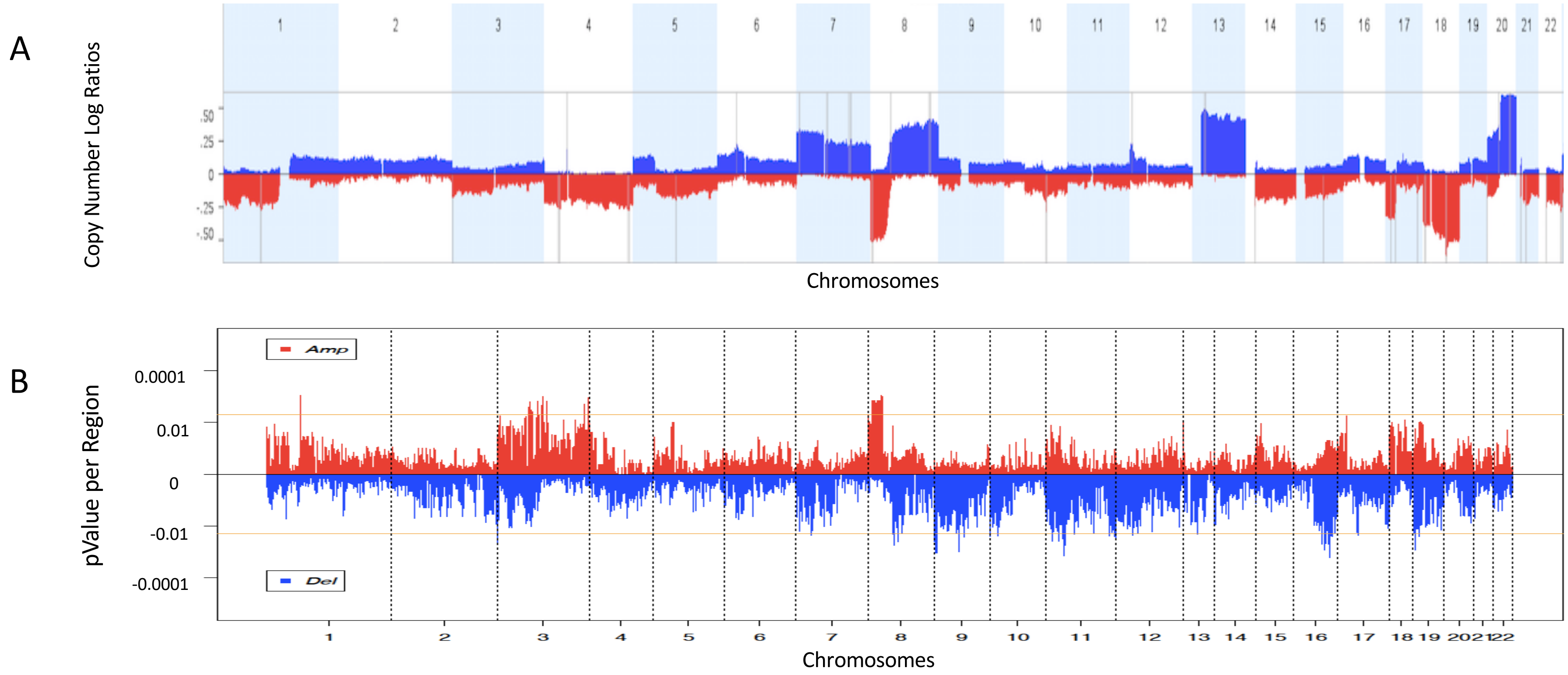

Supplementary Figure 3: Aggregates copy number aberrations of 119 liver metastastic samples and association analysis with PFS in 74 bevacizumab patients.

(A) Aggregate plot displays copy number log ratio of 119 LM samples from 119 patients. The plot above the 0 line is for the gain aggregates and that below 0 is for the loss aggregates. The aggregates are scaled to 1/# of samples so that if all samples participate, the value corresponds to the average value at that location. The gray shading represents the significant GISTIC Regions.

(B) Permutated p-value associated with CN gains (positive value, red) or CN losses (negative values, blue) (y-axis) derived from log-rank tests performed using the PFS data collected from 74 patients treated bevacizumab are plotted as a function of chromosomal position (x-axis). Horizontal yellow lines represent the significance threshold (permutated p-value < 0.005) and vertical dotted lines represent chromosomes boundaries.

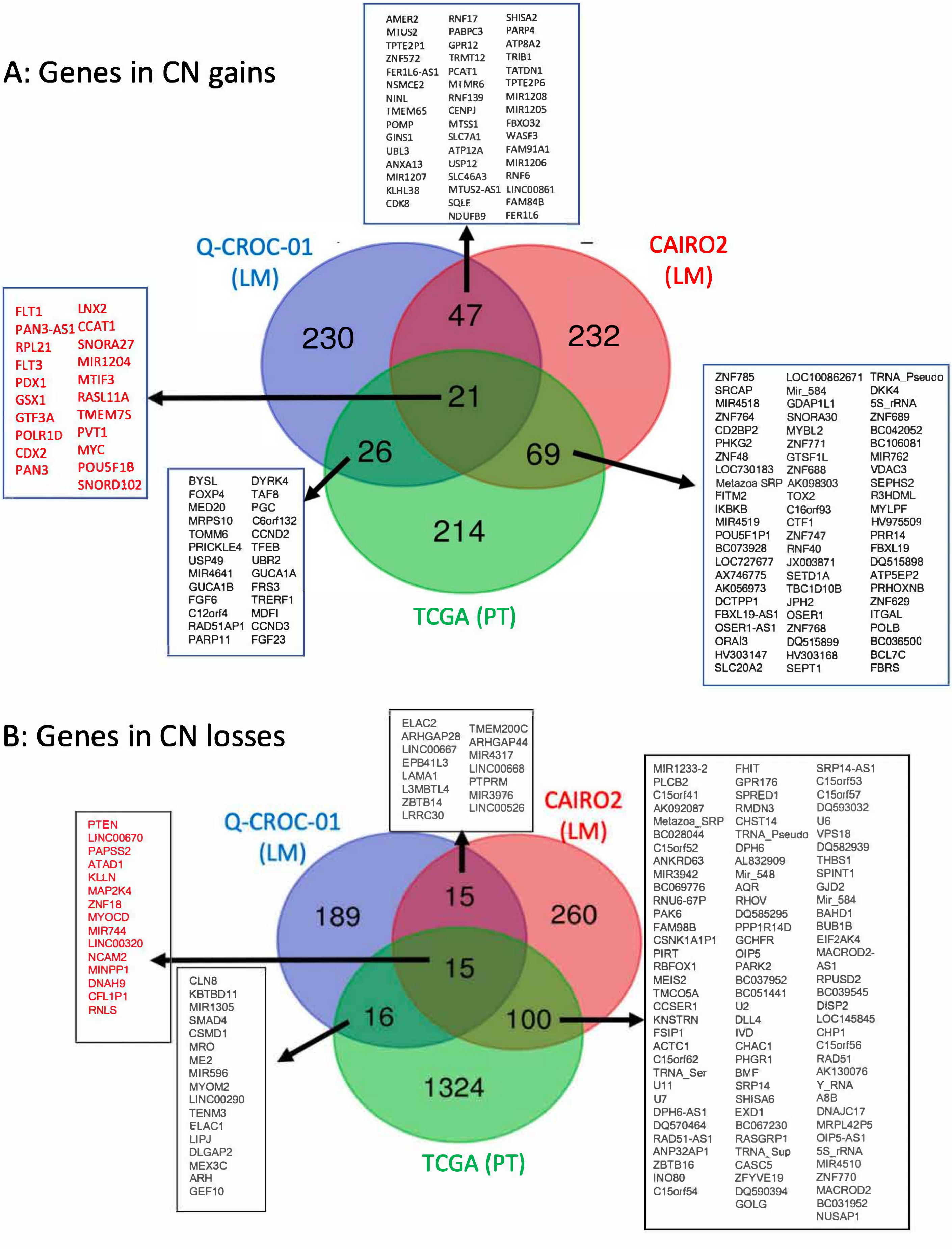

Supplementary Figure 4: Venn diagrams illustrating intersections of gene lists in CN events identified using GISTIC in Q-CROC-01, CAIRO2 and TCGA cohorts. (A): Genes in CN gains. (B) Genes in CN losses.

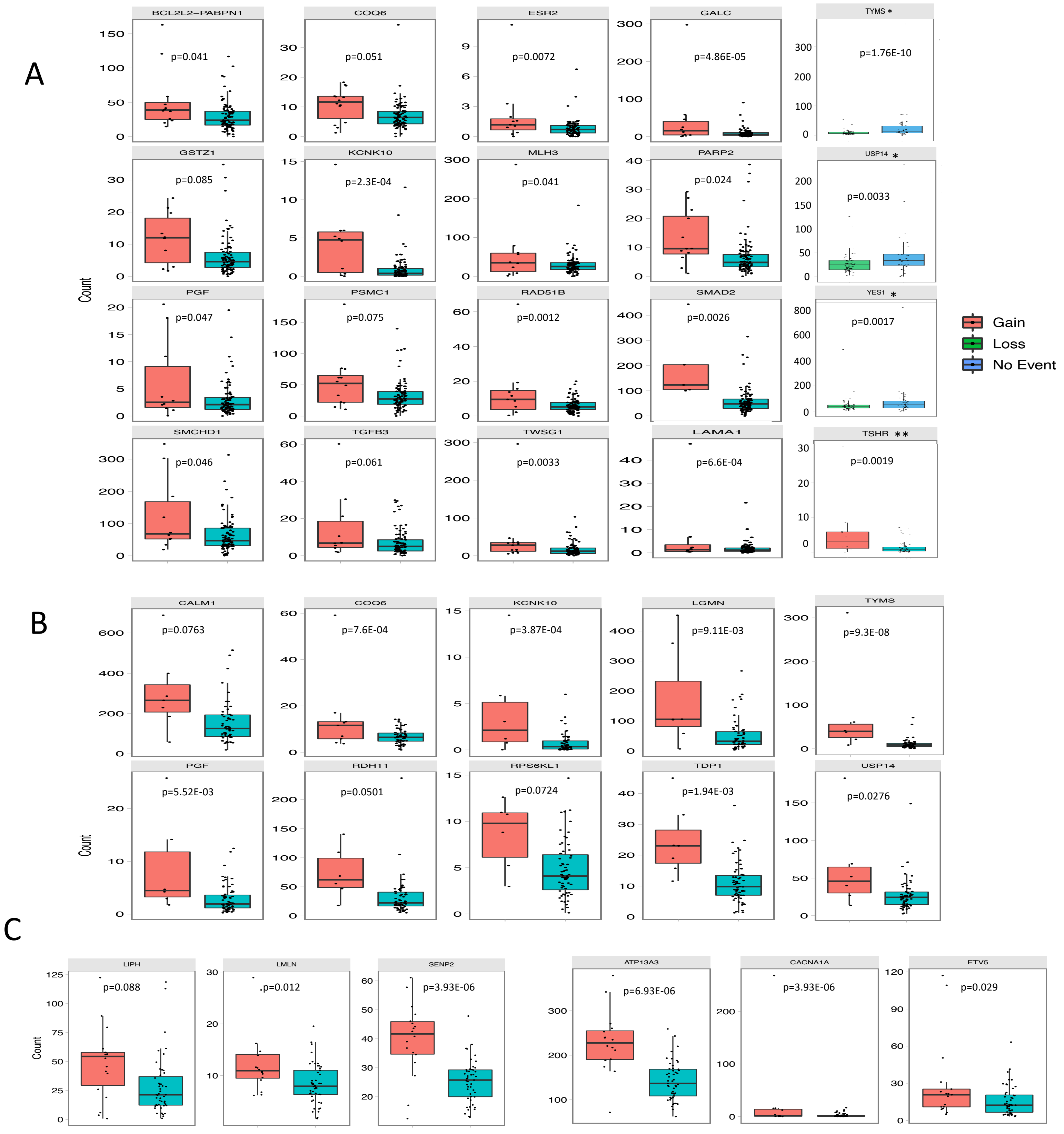

Supplementary Figure 5: Correspondence between copy number change and gene expression in selected candidates identified in group comparison and association analyses. Box plots inferred from RNAseq data showing the impact of copy number changes on the expression (count value) of selected genes lying in CN aberrations found significantly different in frequency between post and pre-treatment samples (A), in post-resistant versus pre-treatment samples (B) and in the association study between CNA and PFS (C). p: FDR adjusted p-value. *Expression values for samples with CN gain are shown in the Figure 2. **Candidate among the 231 cosmic genes.

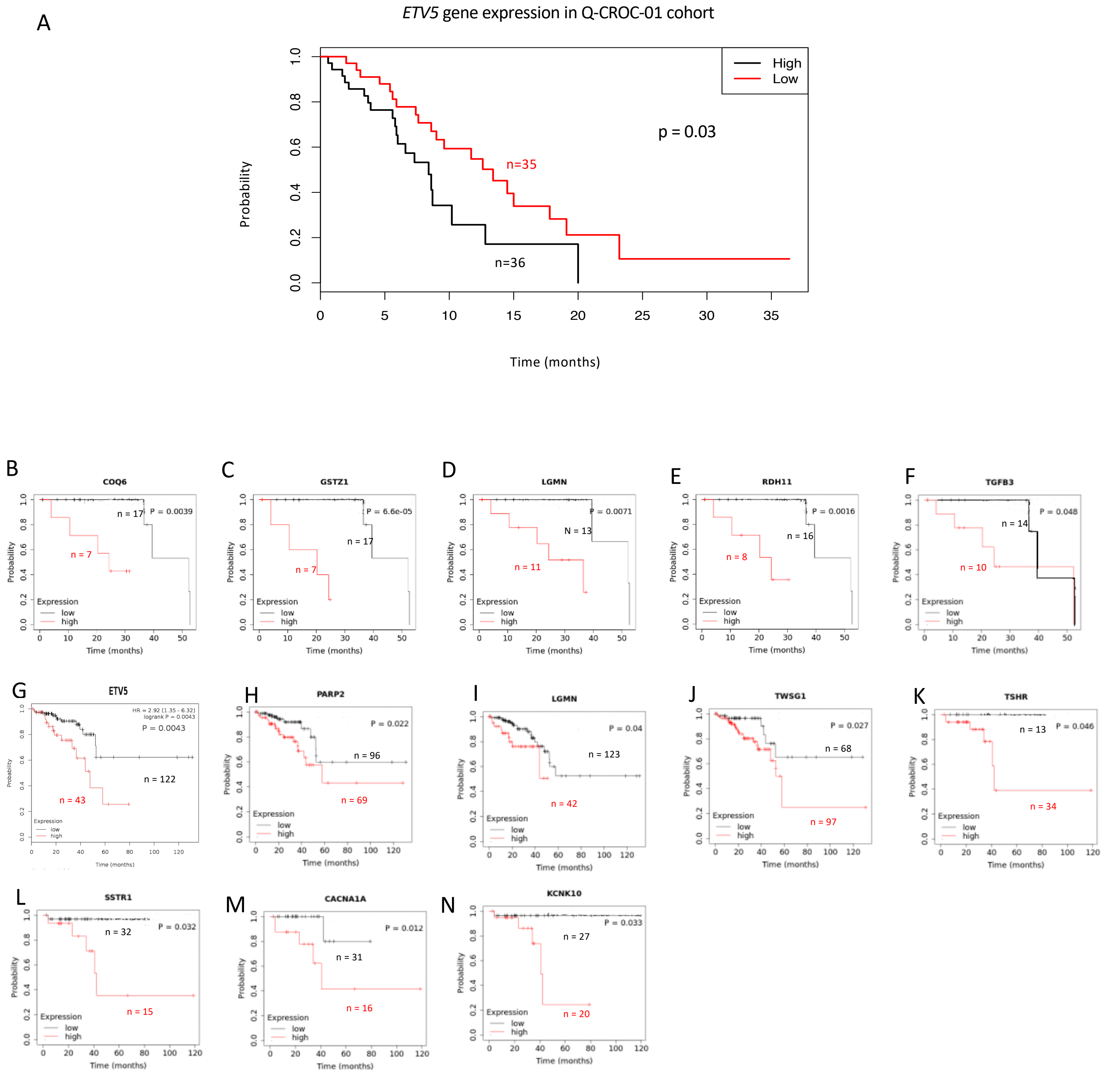

Supplementary Figure 6: Kaplan Meier analyses of survival for high expression versus low expression of selected gene candidates in our QCROC-01 cohort and in Kaplan Meier Plotter database of adenocarcinomas.

(A) High expression versus low expression (based on the median) of *ETV5* was tested for their association with PFS in Q-CROC-01 liver metastasis cohort. (B-F) Association between OS and gene expression level of *COQ6, GSTZ1, LGMN, RDH11 and TGFB3* was shown by Kaplan Meier analysis in 24 stage IV rectum adenocarcinomas. (G-J) Association between OS and gene expression level of *ETV5, PARP2, LGMN, and TWSG1* was shown by Kaplan Meier analysis in 165 rectum adenocarcinomas (all stages). (K-N) Association between RFS and gene expression level of *TSHR*, *SSTR1*, *CACNA1A* and *KCNK10* was shown by Kaplan Meier analysis in 47 rectum adenocarcinomas (all stages).
